# Long-lived and disorder-free charge transfer states enable endothermic charge separation in efficient non-fullerene organic solar cells

**DOI:** 10.1038/s41467-020-19332-5

**Published:** 2020-11-05

**Authors:** Ture F. Hinrichsen, Christopher C. S. Chan, Chao Ma, David Paleček, Alexander Gillett, Shangshang Chen, Xinhui Zou, Guichuan Zhang, Hin-Lap Yip, Kam Sing Wong, Richard H. Friend, He Yan, Akshay Rao, Philip C. Y. Chow

**Affiliations:** 1 Cavendish Laboratory, University of Cambridge, Cambridge, CB3 0HE UK; 2 Department of Physics, The Hong Kong University of Science and Technology, Clear Water Bay, Hong Kong, China; 3 Department of Chemistry, The Hong Kong University of Science and Technology, Clear Water Bay, Hong Kong, China; 4 Institute of Polymer Optoelectronic Materials and Devices, State Key Laboratory of Luminescent Materials and Devices, South China University of Technology, Guangzhou, 510640 China; 5 Department of Mechanical Engineering, The University of Hong Kong, Pokfulam, Hong Kong, China

**Keywords:** Solar cells, Molecular electronics

## Abstract

Organic solar cells based on non-fullerene acceptors can show high charge generation yields despite near-zero donor–acceptor energy offsets to drive charge separation and overcome the mutual Coulomb attraction between electron and hole. Here, we use time-resolved optical spectroscopy to show that free charges in these systems are generated by thermally activated dissociation of interfacial charge-transfer states that occurs over hundreds of picoseconds at room temperature, three orders of magnitude slower than comparable fullerene-based systems. Upon free electron–hole encounters at later times, both charge-transfer states and emissive excitons are regenerated, thus setting up an equilibrium between excitons, charge-transfer states and free charges. Our results suggest that the formation of long-lived and disorder-free charge-transfer states in these systems enables them to operate closely to quasi-thermodynamic conditions with no requirement for energy offsets to drive interfacial charge separation and achieve suppressed non-radiative recombination.

## Abstract (web summary)

Designing efficient organic solar cells is limited by the energy required to overcome the mutual Coulomb attraction between electron and hole. Here, the authors reveal long-lived and disorder-free charge-transfer states enable efficient endothermic charge separation in non-fullerene systems with marginal energy offset.

## Introduction

**Fig. 1:**
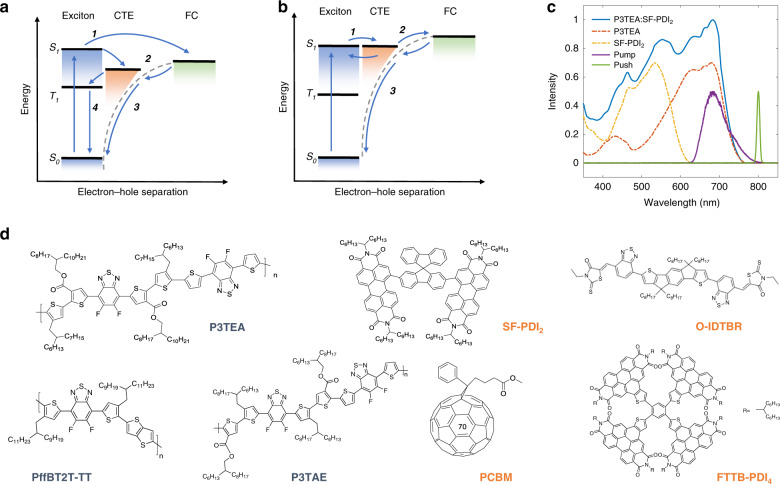
Understanding charge separation processes in highly efficient non-fullerene organic solar cell (OSC) with small photovoltage loss. **a**, **b** Schematic energy diagram illustrating the time evolution of charges in **a** conventional fullerene and **b** non-fullerene OSC systems. Conventional fullerene systems are designed with large donor–acceptor (D/A) offsets and generally have high degree of electronic disorder at the interface (Urbach energy ≥ 40 meV), whereas non-fullerene systems are designed with small offsets and generally have low disorder (Urbach energy ~25–30 meV). Photoexcited states evolve through the following three stages: (1) dissociation of singlet excitons into CTEs or directly into free charges, (2) separation of CTEs into free charges, and (3) charge recombination. Additional losses through triplet exciton formation is often found for fullerene systems (process 4). **c** Absorption spectra of pristine P3TEA and SF-PDI_2_, and P3TEA:SF-PDI_2_ blend on quartz substrate. We preferentially create photoexcitations in P3TEA (pump at 670 ± 40 nm) for all sub-nanosecond transient absorption measurements presented in this work. For pump–push–probe measurements, a push pulse (time-delayed to the pump) arrives to interact with excited states and create a characteristic optical response that is subsequently monitored by a broadband probe pulse. The push pulse has an energy below the optical gap (push energy = 800 ± 10 nm) and does not generate more photoexcitations directly from the ground state (see Supplementary Information). **d** Chemical structure of donor polymers (P3TEA, P3TAE, and PffBT2T-TT) and acceptor molecules (SF-PDI_2_, O-IDTBR, FTTB-PDI_4_, and PCBM) involved in this study. Additional material and device characterizations are found in the Supplementary Information.

Organic solar cells (OSCs) are flexible, semitransparent and environmental-friendly alternatives to inorganic solar cells. In contrast to inorganic semiconductors, photoexcitation of organic semiconductors generates coulombically bound electron–hole pairs known as excitons^1^. These excitons have large binding energies (typically ~0.5 eV) and do not separate into free charges unless they are dissociated at a donor/acceptor (D/A) heterojunction to form charge-transfer excitons (CTEs), as shown in Fig. 1a, b, which must subsequently separate to free electrons and holes against their mutual Coulomb interaction in order to create photocurrent. Bulk-heterojunction OSC blends with nanoscale morphology are able to achieve nearly 100% free charge generation yields^2^. However, their overall power conversion efficiency (PCE) is limited by the relatively low open-circuit voltage (*V*_OC_) with respect to their optical gap (*E*_*S*1_). Current models of operation in OSCs introduce three sources of photovoltage (energy) losses. The first is losses due to photon entropy increase, which are unavoidable in all types of solar cells (typically ~0.3 V in the Shockley–Queisser framework)^3^. The second is non-radiative recombination of charge pairs, either by phonon mediated relaxation to the ground state^4^ or formation of low-energy spin-triplet excitons which subsequently relax to the ground state^5,6^. The magnitude of the non-radiative voltage loss is given by $V_{\rm{non-rad}} = - kT {\rm{ln}}\left( {{\rm{EQE}_{EL}}}\right)/q$, where *k* is the Boltzmann constant, *T* the absolute temperature, *q* the elementary charge, and EQE_EL_ the EL quantum efficiency^7^. The third source of photovoltage loss is the offset between the energy of the photogenerated singlet excitons (*E*_*S*1_) and the interfacial CTEs (*E*_CTE_), see Fig. 1a. This energy offset, *E*_*S*1_–*E*_CTE_, is widely believed to be required in order to drive efficient and rapid (within a few hundred femtoseconds) charge separation at organic heterojunctions^2,8,9^.

Previous studies have shown that a large *E*_*S1*_–*E*_CTE_ offset (~200 meV and above) is typically required to drive charge separation in conventional OSCs that use fullerene acceptors (such as [6,6]-phenyl-C_71_-butyric acid methyl ester or PCBM). This offset requirement in turn leads to the formation of CTEs with energy below the optical gap, as evidenced by sub-gap emission and absorption tails of the blends^10^. In addition, CTEs in fullerene systems often introduce energetic disorder that broadens the density of states (Urbach energy typically ≥40 meV for fullerene blends compared to ~25–30 meV for pure organic materials) that can increase charge recombination^11^. We note that there exist several examples of fullerene OSCs that can achieve decent performance with small offsets^12–14^; however, most fullerene systems engineered with small offsets show reduced charge generation quantum yields^15–17^. Furthermore, fullerene systems typically give EQE_EL_ ≤ 10^−6^, resulting in a typical non-radiative recombination loss of >0.35 V^18^. Overall, the combination of these terms results in photovoltage losses in the range of ~0.7–1.0 V with respect to *E*_*S*1_/*q* for fullerene OSCs with large energy offsets^19,20^.

Alternatively, many recent examples of OSC systems based on non-fullerene acceptors show high charge generation yields with negligible *E*_*S*1_*–E*_CTE_ offsets (≤0.05 eV; see Fig. 1b), and high EQE_EL_ (≥10^−4^)^14,21–27^. The combination of low *E*_*S*1_–*E*_CTE_ offsets and high EQE_EL_ results in greatly reduced photovoltage losses (≤0.6 V), and state-of-the-art materials with broad spectral overlap with sunlight can now achieve over 17% PCE in single-junction devices^28^. Considerable effort has been directed toward exploring charge separation process within this material class, with recent studies exploring the roles of exciton-CT state hybridization^29–32^, molecular vibrations^33^, electrostatic interactions^34,35^, electronic delocalization^36^ among other properties. However, the precise mechanism and dynamics of electron–hole separation in these efficient OSC systems have remained unclear.

Here, we study the charge separation dynamics in a series of non-fullerene OSC systems with small photovoltage losses and high charge generation quantum yields. First, we use two-pulse (pump–probe; PP) transient absorption spectroscopy to probe the conversion of photogenerated excitons to CTEs and free charges. Then, we use three-pulse (pump–push–probe; PPP) transient absorption to selectively monitor the population of CTEs confined at the donor–acceptor interface. The combination of these optical methods allows us to obtain a detailed overview of the excited state dynamics as a function of time and temperature, revealing how free charges efficiently separate with suppressed non-radiative recombination losses in non-fullerene OSC systems with small energy offsets.

## Results

### Material systems

Our study involves four model non-fullerene blends, namely P3TEA:SF-PDI_2_^24^, P3TEA:FTTB-PDI_4_^37^, P3TAE:SF-PDI_2_^38^, and PffBT2T-TT:O-IDTBR^39^. Information about their chemical structures, absorption spectra, energy levels, blend morphologies, and photovoltaic performances can be found in Fig. 1c, d and in the Supplementary Information (Supplementary Figs. 2–4). Among these we focus on the P3TEA:SF-PDI_2_ blend. With an *E*_*S*1_ of 1.72 eV (determined by the intercept of absorption and emission spectra), the large *V*_OC_ of 1.11 V indicates a photovoltage loss of 0.61 V. Of this only ~0.25 V is due to non-radiative recombination losses as determined by the EQE_EL_ of 0.5 × 10^−4^. Fitting of the absorption tail and EL spectrum places the CTE energy at ~1.7 eV (see Supplementary Fig. 4), indicating a negligible energy offset between *E*_*S*1_ and *E*_CTE_, as shown in Fig. 1b. The steep absorption edge shows that CTEs have low degree of electronic disorder (Urbach energy of ~27 meV). Despite the small *E*_*S*1_–*E*_CTE_ offset, P3TEA:SF-PDI_2_ devices exhibit photovoltaic internal quantum efficiency (IQE) of around 85% at short-circuit. The relatively modest PCE of 9.5% compared to other state-of-the-art systems is mainly due to its weak absorption of sunlight in the near-infrared region. For comparison, we contrast this system with its less efficient fullerene-based counterpart: P3TEA:PCBM (PCE of 7.5%, with a photovoltage loss of 0.83 V). The CTEs in the fullerene blend has energy of ~1.6 eV (see Supplementary Fig. 4), giving an *E*_*S*1_–*E*_CTE_ offset of about 100 meV, and have low electronic disorder comparable to those found in the non-fullerene blend. We observe strong photoluminescence (PL) quenching for both blends with respect to the pure polymer film, thus indicating efficient dissociation of excitons into charges at D/A interfaces (see below for more discussion). Negligible *E*_*S*1_–*E*_CTE_ offsets and low CTE disorder are also found in P3TEA:FTTB-PDI_4_, P3TAE:SF-PDI_2_, and PffBT2T-TT:O-IDTBR blends. Devices based on these blends can achieve large *V*_OC_ of 1.13, 1.19 and 1.08 V, respectively. The combination of small voltage losses (<0.6 V) and high external quantum efficiencies (>60%) allows these systems to achieve high PCEs despite their weak absorption in the near-infrared (see Supplementary Fig. 2). As can be seen from Fig. 1d, the various non-fullerene acceptors cover a range of different structural motifs and allow us to generalize the results of our study to any non-fullerene system with marginal energy offsets to drive charge separation.

### PP transient absorption spectroscopy

**Fig. 2:**
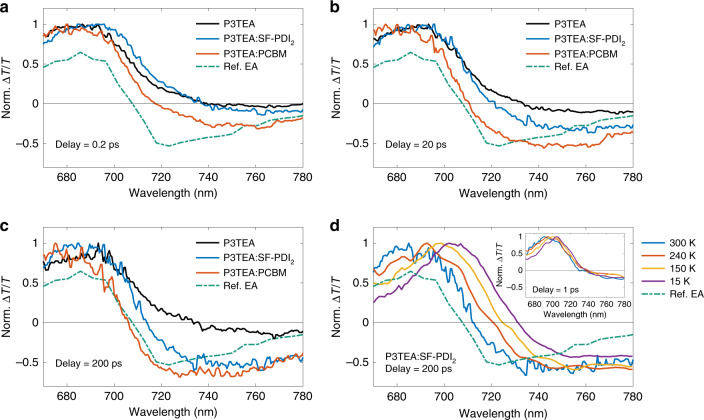
Monitoring charge separation dynamics using pump–probe (PP) spectroscopy. **a**–**c** Normalized PP transient absorption response (∆*T*/*T*) for pristine P3TEA, P3TEA:SF-PDI_2_, and P3TEA:PCBM near the absorption edge at room temperature (photoexcited at 670 nm, 1.5 µJ cm^−2^ per pulse). Non-normalized data over full detection range can be found in Supplementary Figs. 8 and 9. At early times (0.2 ps; panel **a**), the non-fullerene blend shows similar PP response to the pristine polymer which is associated with photogenerated excitons, while a negative ∆*T*/*T* signal from ~720 nm onwards associated with charges (hole polarons) has already emerged for the fullerene blend. As detailed in the main text, this negative signal is due to the growth of a transient electroabsorption (EA) signal caused by charge separation across the D/A interface as well as polaron absorption. With increasing time delay (20 ps; panel **b**), a similar negative ∆*T*/*T* signal emerges in the non-fullerene blend, but not evolving to match the fullerene ∆*T*/*T* signal until ~200 ps (panel **c**) at which both signals are similar to the quasi-steady-state EA response measured in a diode structure (Ref. EA). This indicates that charge separation in the non-fullerene blend takes much longer time to complete compared to the fullerene blend. **d** PP response of P3TEA:SF-PDI_2_ at reduced temperatures. At early time (1 ps; inset) the response (associated mostly with singlet excitons) is largely unchanged with respect to that of the pristine polymer (see Supplementary Fig. 10). We note that the slight spectral red-shift (≤20 nm) of the ground-state bleach with reduced temperature is due to lowering of the optical gap. At later times (~200 ps), we find much weaker signs of transient EA signal created by charge separation at reduced temperatures (as evidenced by the much reduced spectral blue-shift), thus implying that it is indeed an endothermic process. In contrast, reducing temperature has insignificant effect on charge separation in the fullerene blend, where a clear EA signal is seen even at ~20 K (see Supplementary Fig. 10).

We first monitor excited state dynamics using a broadband white-light pulse to probe the films following excitation with a pump pulse (spectrum shown in Fig. 1c). Figure 2a–c compares the PP spectra of pristine P3TEA, P3TEA:SF-PDI_2_, and P3TEA:PCBM films near the absorption edge (670–780 nm) at various PP time delays (non-normalized spectra over the full detection range can be found in Supplementary Figs. 8 and 9). Photoexcitation of the pristine polymer film leads to the formation of singlet excitons and we observe a positive ∆*T*/*T* signal up to ~740 nm that matches the polymer absorption (thus corresponding to the ground-state bleach; GSB). The PP response of the pristine polymer does not evolve spectrally up to 1 ns, indicating that singlet excitons are the only excited state species. At early times (0.2 ps; Fig. 2a), the non-fullerene blend (P3TEA:SF-PDI_2_) shows a similar PP response to the pristine film, thus indicating that singlet excitons make up most of the excited states. In contrast, the fullerene (P3TEA:PCBM) blend already shows a negative ∆*T*/*T* signal emerging at ~720 nm onwards that is similar to the quasi-steady-state electroabsorption (EA) response measured in a diode structure. This negative ∆*T*/*T* signal is not found for the pristine polymer and is thus attributed to charges (polarons) created at the D/A interface from very early times^2^. More specifically, this negative ∆*T*/*T* signal can be explained by two partially overlapping features. We attribute the signal at longer wavelengths (>750 nm) to polaron absorption and the signal around 720 nm, which leads to a blue-shift of the absorption edge, to a transient EA response. As discussed in detail in previous work^2,8^, the transient EA response arises from a Stark shift of the absorption spectrum due to the local electric fields generated between electron–hole pairs following charge separation at the D/A heterojunction. The emergence of this transient EA response in PP measurements is thus a signature of the charge separation process (see Supplementary Information for a detailed description). Our results indicate that the formation of free charges (spatially separated) occurs on an ultrafast timescale (<1 ps) in the fullerene blend, which is consistent with previous measurements on other fullerene-based systems with large D/A energy offsets.

**Fig. 3:**
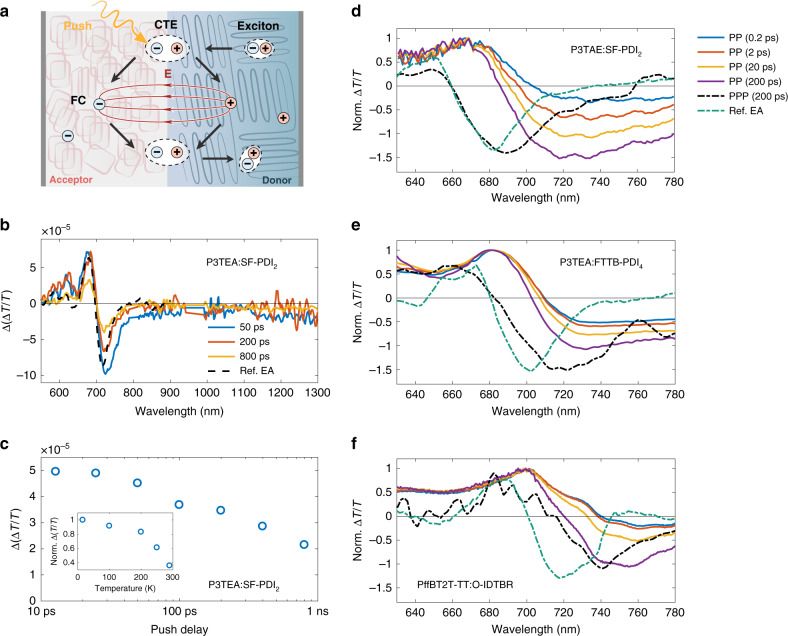
Tracking charge transfer excitons (CTE) using pump–push–probe (PPP) spectroscopy. **a** Schematic diagram of the excited state dynamics at the donor–acceptor heterojunction. If the push is absorbed by a CTE state it increases charge separation, causing an electroabsorption (EA) response near the absorption edge. **b** Change in transient absorption response (∆(∆*T*/*T*)) for P3TEA:SF-PDI_2_ measured at various pump–push time delays (after subtraction of effects due to annihilation of excitons, see Supplementary Information). The signal near the absorption edge (~700 nm) closely resembles the quasi-steady-state EA response measured in a diode structure (Ref. EA) and can therefore be attributed to the push-induced response of bound CTE population. The pump (670 nm) and push (800 nm) pulses have a fluence of 50 µJ cm^−2^ and 35 µJ cm^−^^2^ per pulse, respectively. **c** Evolution of the CTE signal (absolute value of signal between 640 and 740 nm), showing a substantial amount of CTEs still present at long time (~800 ps). The inset shows the temperature dependence of CTE signal per photogenerated singlet exciton at 50–100 ps. At lower temperatures the CTE population increases, consistent with endothermic charge separation. **d**–**f** Pump–probe (PP) and pump–push–probe (PPP) spectra of P3TAE:SF-PDI_2_, P3TEA:FTTB-PDI_4_, and PffBT2T-TT:O-IDTBR (sharing the same legend). All blends show a growing charge signal in the PP spectra (negative ∆*T*/*T* response near the absorption edge) and an EA response in the PPP spectra at 200 ps (compared here to the derivative of the PP signal of the pristine polymer at 1 ps; labeled as Ref. EA).

With increasing time delay (Fig. 2b, c), a similar transient EA response emerges in the non-fullerene sample at ~20 ps, but does not evolve to match the response of the fullerene blend until ~200 ps. Remarkably, the timescale at which the transient EA response emerges for the non-fullerene system shows that long-range charge separation occurs at a much slower rate compared to the fullerene blend, taking over ~100 ps to complete. Similar charge separation times are also found in P3TEA:FTTB-PDI_4_, P3TAE:SF-PDI_2_, and PffBT2T-TT:O-IDTBR (see Fig. 3d–f). We highlight that by tracking the growth of the transient EA signal we monitor the separation dynamics of excitons/CTEs into free charges, while other reports to date focused on measuring the charge transfer time across the D/A heterojunctions (i.e., dissociation of excitons into CTEs) in non-fullerene OSCs^29,40–42^.

The slow timescale for charge separation of CTEs measured here (~100 ps) contrasts with the fast rate of vibrational relaxation for excitons and CTEs (~100 fs)^1^. This means that CTE separation must occur from thermally relaxed CTEs^43^. We note that no “excess energy” from the donor–acceptor offset is available to drive charge separation for these systems (negligible *E*_*S*1_–*E*_CTE_ offsets). However, as the internal energy of free charges must lie above the coulombically bound CTE state (see Fig. 1b), it follows that charges must overcome the Coulomb energy barrier to separate into free charges. This barrier, the CTE binding energy, is typically found to be 200–250 meV in organic blends^2,11,44^. We thus propose that the slow charge separation measured here for the non-fullerene systems is due to the need for thermal activation of the CTEs to free charges, making the process endothermic.

To explore this further we perform PP spectroscopy at reduced temperatures. For pristine P3TEA, we find a slight red shift (≤20 nm) of the GSB due to lowering of the optical gap upon cooling (see Supplementary Fig. 10). For the non-fullerene blend at early times (~1 ps; inset of Fig. 2d), the PP response (associated mostly with singlet excitons) is largely unchanged with respect to that of the pristine polymer. However, as shown in Fig. 2d, we do observe a clear effect of temperature on the PP response at later times (~200 ps), showing much weaker signs of transient EA signal created by charge separation at reduced temperatures (as evidenced by the much reduced spectral blue-shift). This indicates that long-range charge separation in the non-fullerene blend is suppressed at lower temperatures, providing evidence that it is indeed an endothermic process. In contrast, reducing temperature has insignificant effect on long-range charge separation in the fullerene blend, where a clear EA signal is seen even at ~20 K (see Supplementary Fig. 10). This result is consistent with the considerable *E*_*S*1_–*E*_CTE_ offset measured for this system (see Fig. 1a and Supplementary Fig. 4) and agrees with previous reports that charge separation via the delocalized electronic states of fullerene acceptors is a temperature-independent process^2,45^.

When examining the effect of temperature on the charge generation yield, we find that almost no photocurrent is generated below ~120 K for the P3TEA:SF-PDI_2_ device (see Supplementary Fig. 5). In comparison, we find that P3TEA:PCBM has a much weaker dependence on temperature. Weak temperature dependence of photocurrent yield is also found in other efficient fullerene-based systems that are designed with energy offsets to drive charge separation^45^. These results are consistent with the proposed endothermic nature of free charge generation in the non-fullerene blends studied here. We note that the activation energy determined by fitting of the steady-state photocurrent at various temperatures does not provide a direct measure of the Coulomb binding energy of CTEs, but instead provides a measure of the interplay between charge dissociation and recombination rates at various temperatures (see below for more discussion).

### PPP transient absorption spectroscopy

While PP spectroscopy allows us to track the spatial separation of charges via growth of the transient EA signal, it does not provide a picture of the size of CTE population confined at the heterojunctions. To observe this, we turn to PPP spectroscopy. In this technique the sample is excited twice: an above-gap “pump” pulse generates an excited state population, which is then influenced by a ‘push’ pulse. The push wavelength is selected so that it is not absorbed by the ground state, as shown in Fig. 1c, but can be absorbed by excited states (excitons, CTEs and free charges). The optical response to this perturbation is then recorded by a third, broadband probe pulse. For the P3TEA:SF-PDI_2_ blend, absorption of the “push” by excitons leads to annihilation of excited states, whereas absorption by free holes causes no change in the spectrum. However, when absorbed by CTE states, the push causes an increase in electron–hole separation, as illustrated in Fig. 3a, which we observe as an increase in the EA response^11,46^. Figure 3b shows the PPP response of P3TEA:SF-PDI_2_ for several pump–push delay times with a constant push–probe delay of 1 ps. Here, we have subtracted the ‘annihilation’ component from exciton absorption to reveal the effect of the push pulse on the CTE states (see Supplementary Information for details on this procedure). Also shown is the quasi-steady-state EA response measured in a diode structure which matches the PPP spectrum near the absorption edge (~700 nm), showing that the push pulse indeed separates CTE states and gives rise to an EA response. By tracking the strength of this PPP response, we are thus able to monitor the population of coulombically bound CTEs confined at the heterojunctions.

Figure 3c shows the evolution of this PPP spectral response in the P3TEA:SF-PDI_2_ blend. We observe a drop in CTE population between ~10 and 100 ps, which agrees with the PP data (Fig. 2) that show long-range charge separation occurring on this timescale at room temperature. However, the CTE population does not fall to zero, with a substantial CTE signal still present at 800 ps. From the PP data, we know that there is no growth in EA beyond 200 ps, indicating that the population of free charges does not substantially increase beyond this point. We know that free charge generation is very efficient in this blend, with IQE of ~85%, so that the long-time CTE signal is mostly associated with CTEs that do eventually separate to free charges. We have noted above that the slow charge separation we observe here occurs from thermalized CTEs, which will also be the states populated via bimolecular encounters of free electrons and holes. We consider therefore that this long-lived PPP signal arises from CTE formed at long timescales due to bimolecular charge encounters. Thus, we observe here the build-up of a quasi-equilibrium between free charges and CTEs, in agreement with previous suggestions in the literature^14,44^.

At lower temperatures we find the PPP response in the P3TEA:SF-PDI_2_ blend increases, indicating an increase in CTE population. The inset of Fig. 3c shows the CTE signal at 50–100 ps after the initial photoexcitation at various temperatures, normalized to the singlet exciton signal. The corresponding spectra are shown in Supplementary Fig. 12. This indicates that the temperature dependence of charge separation observed in PP measurements (Fig. 2) is not due to trapping of excitons caused by morphology change at reduced temperature, but instead due to the endothermic nature of CTE separation in the non-fullerene system. The substantial CTE signal still present at long time (~800 ps) suggests that within these systems the quasi-equilibrium favors the bound CTE states in the absence of charge extraction, i.e., open-circuit conditions. Without a thermally activated charge separation channel (which we observe both after initial photoexcitation and following free electron–hole encounters), this would lead to poor device IQEs.

For P3TAE:SF-PDI_2_, P3TEA:FTTB-PDI_4_, and PffBT2T-TT:O-IDTBR, we also observe a long-lived PPP spectral response with derivative line-shape near the optical edge that we attribute to CTEs at the D/A heterojunction of these blends (see Fig. 3d–f). These CTE signals do not fall to zero beyond the free charge generation timescale (up to 800 ps; see Supplementary Fig. 14), in agreement with our observations for the P3TEA:SF-PDI_2_ blend of a quasi-equilibrium between CTEs and free charges. Overall, we observe very similar PP and PPP results in the four efficient non-fullerene blends with small photovoltage losses despite their different structural motifs, thus showing that the slow (tens to hundreds of ps) yet efficient endothermic charge separation process is general to non-fullerene systems with near-zero *E*_*S*1_–*E*_CTE_ offsets. In contrast, we observe a very weak PPP response in P3TEA:PCBM at ~200 ps (see Supplementary Fig. 13), thus indicating that most charges are spatially separated and few CTEs are present.

### Charge recombination dynamics

**Fig. 4:**
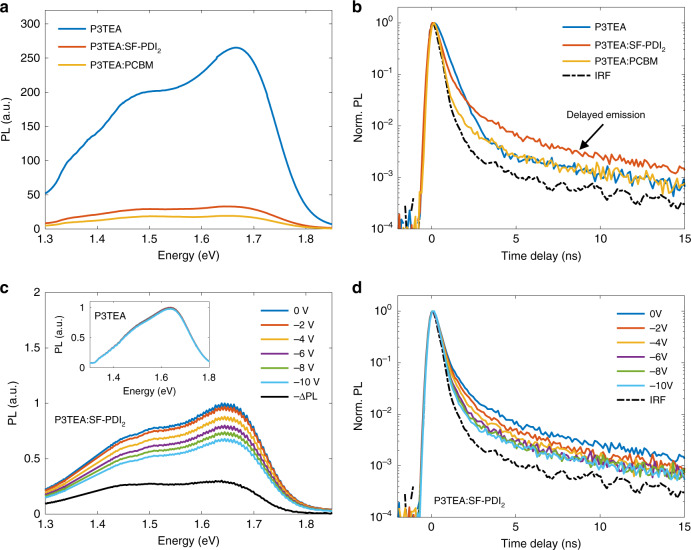
Regeneration of emissive singlet exciton states upon free electron–hole encounters. **a** Time-integrated PL spectra of pure P3TEA, P3TEA:SF-PDI_2_, and P3TEA:PCBM thin-films under 633 nm excitation (corrected for difference in absorbance between samples). Using an integrating sphere we estimated a PL quantum yield of ~2% for the pure polymer film, ~0.2% for the non-fullerene film and ~0.1% for the fullerene film. **b** Time-resolved PL decay of thin-film samples under pulsed 680 nm excitations. A 780 nm (1.58 eV) long-pass filter was used to define the detection wavelength range. Additional measurements with a specific detection wavelength at ~750 nm (1.65 eV) show similar decay kinetics for the non-fullerene sample (see Supplementary Fig. 6). Initial PL quenching at early times reflect efficient exciton dissociation in both blends, but delayed emission on a nanosecond timescale is observed only for the non-fullerene blend (consistent with the higher PL quantum yield). **c** Time-integrated PL of P3TEA:SF-PDI_2_ device at various applied bias conditions, showing reduced PL with increasing reverse bias. The black line −∆PL (PL at 0 V minus PL at −10 V) corresponds to the emission that arises from charge recombination events (i.e., reverse bias suppresses free electron–hole encounters). The inset shows PL of a pure P3TEA device taken over the same bias range, showing negligible differences. **d** Time-resolved PL decay of P3TEA:SF-PDI_2_ under various bias conditions (same excitation and detection conditions as in panel **b**). The reduction of delayed emission by applied reverse bias indicates that emissive singlet states are regenerated upon free electron–hole encounters. IRF instrument response function.

We now turn to the charge recombination dynamics. As shown in Fig. 4a, c, the PL spectral shapes of pristine P3TEA and P3TEA:SF-PDI_2_ films are similar despite the large difference in intensity (PL quantum yield of ~2% and ~0.2%, respectively). Figure 4b shows time-resolved PL measurements, where following the initial PL quenching due to dissociation of excitons, we observe a delayed emission on a nanosecond timescale for the non-fullerene systems. Importantly, this delayed emission is longer lived than the PL of the pristine material and is not observed for the fullerene blend (PL quantum yield of ~0.1%). This suggests that it is due to radiative recombination of long-lived CTEs found in the non-fullerene blend.

We further study the PL of these materials in a diode structure. As shown in Fig. 4c, we find that the PL intensity of the non-fullerene blend decreases uniformly with applied reverse bias, whereas no difference is observed for the pristine polymer device over the same bias range (see inset). This indicates that, while the applied electric field is insufficient for separating excitons in the pure material, in the blend it causes the free electrons and holes to drift towards opposite electrodes and thus reduces the radiative charge recombination^47^. Therefore, we obtain the emission spectrum arising from charge recombination by taking the differential PL spectrum of the device (PL at short-circuit minus PL with reverse bias of 10 V). We find that this differential emission (–ΔPL) closely resembles the PL spectrum of the pristine material. Since the CTE energy is close to that of the singlet exciton of the donor polymer (Fig. 1b), it is very likely that the emission caused by charge recombination is from singlet excitons that are populated by electron back-transfer from CTEs^29–31^. Furthermore, as shown in Fig. 4d, we find that the application of reverse bias suppresses the aforementioned delayed emission observed in the non-fullerene blend (consistent with the reduced PL intensity).

We thus assign this bias-dependent, nanosecond PL to singlet excitons formed via CTEs that are regenerated upon bimolecular encounters of free electrons and holes at the D/A heterojunction. This is consistent with the response observed at longer times in the PPP measurements (Fig. 3) and implies that the quasi-equilibrium includes the singlets excitons as well as CTEs and free charges, allowing for excitations to cycle between the states until they recombine to the ground state via the singlet exciton state (CTE to ground state transition is much slower^29,30^). This indicates that the charge separation yield can be limited by fast singlet recombination, and thus explains the correlation between open-circuit voltage and PL quantum yield of the low-gap component found in other non-fullerene systems with small offsets^29,31^. Since we show that CTEs take ~100 ps to fully separate at room temperature, the singlet exciton lifetime must exceed this timescale for efficient charge separation to occur^32^. This is consistent with the effective PL quenching found for P3TEA:SF-PDI_2_ despite the regeneration of singlet excitons. The presence of an equilibrium between excitons and charges also implies that the singlet regeneration rate is comparable to the rate at which singlets form CTEs (~1–20 ps according to our PP results, see Fig. 2). Furthermore, it is well-known that the formation of triplet excitons can lead to substantial non-radiative recombination losses in OSC blends. Similar to other fullerene blends^5,6^, our PP transient absorption results for P3TEA:PCBM show clear spectral shifting on the nanosecond timescale that is likely caused by a growth in the polymeric triplet signal (see Supplementary Fig. 11). This is a signature of triplet exciton formation following bimolecular charge encounters (process 4 in Fig. 1a), though additional characterizations are needed to confirm and quantify triplet recombination in this fullerene blend. In contrast, we find no spectral shifting in P3TEA:SF-PDI_2_ over the same timescale, which suggests a suppression of triplet recombination in the non-fullerene blend.

## Discussion

For conventional fullerene-based OSCs with large offsets it has been widely noted that the rate of bimolecular recombination is suppressed compared to Langevin recombination models, and a number of explanations such as phase segregation of electrons and holes have been put forward to explain this^44,48^. However, in these fullerene systems, free electron–hole encounters do not regenerate singlet excitons to any significant extent as there is a substantial energetic offset between the relaxed CTEs (which are formed via bimolecular charge encounters) and the singlet excitons (Fig. 1a). The large energetic offset in turn is required to drive rapid charge separation. We note that thermally activated separation of the relaxed CTEs has been observed for a number of fullerene systems^43^. However, most fullerene systems with reduced offset show lower EQE^15–17^, thus implying that thermally activated separation of the CTEs is not efficient in most systems involving fullerene acceptors. A likely reason for this is the high degree of electronic disorder found in many fullerene blends (Urbach energy ≥40 meV) which traps the CTEs in low-energy sites, from which they cannot escape to form free charges or regenerate singlet excitons due to fast non-radiative recombination via vibrational relaxation^4,30,33^ as well as triplet formation^5,6,49^. Fullerene systems with small offsets that do show decent performance were found to have low electronic disorder (for examples, PIPCP:PCBM^11^ and PNOz4T:PCBM^29^). Thus, due to the requirement of an energetic offset for efficient charge separation in most fullerene-based systems, the EQE_EL_ values for fullerene OSCs are generally very low (≤10^−6^, as measured here for P3TEA:PCBM system).

However, our results presented here indicate that, in the non-fullerene systems with marginal energetic offsets, charge separation occurs at up to hundreds of picoseconds from long-lived CTEs via thermal activation. These same CTEs are repopulated via free electron–hole encounters, allowing for the repopulation of the singlet exciton state. This consequently leads to an equilibrium between the singlet excitons, CTEs, and free charges. This much more efficient regeneration of emissive singlet excitons in the lower-gap material of the blend enables a higher EQE_EL_ and hence lower non-radiative voltage losses. Importantly, with sufficiently long lifetimes, the regenerated singlet excitons can then once again form CTEs that have a high probability of redissociating into free charges at room temperature. We note that the long CTE lifetime in these non-fullerene systems is likely assisted by: (1) the low degree of electronic disorder at their interfaces, which is comparable to thermal energy at room temperature (Urbach energy ~27 meV in P3TEA:SF-PDI_2_, see Supplementary Fig. 4)^11^ and (2) the slow CTE-to-triplet exciton transition rate.

The reversible interconversion of free charges, CTEs and singlet excitons suggest that their Gibbs free energies must be very similar for the non-fullerene OSC systems. This contrasts with conventional fullerene-based systems, where the large D/A energy offset significantly lowers the Gibbs free energy of the free charges with respect to CTEs and singlet excitons, thus introducing irreversibility into the system. This makes conventional fullerene OSC systems similar to natural light harvesting complexes, in which an energy cascade is also used to drive long-range charge separation on ultrafast timescales. This exothermic process leads to near-unity IQE but only in the expense of a large photovoltage loss. However, as we have shown here, highly efficient endothermic charge separation can occur in the non-fullerene blends via thermal activation of long-lived and disorder-free CTEs on long timescales (>100 ps). This endothermic process removes the need for charges to suffer a loss in free energy in order to obtain near-unity IQE and allows for minimal photovoltage loss.

Efficient endothermic charge separation brings non-fullerene OSCs, in terms of their thermodynamics, into the same mode of operation as inorganic solar cells, where encounters of free carriers do not directly lead to terminal recombination (in the absence of non-radiative decay events associated with defect states) and the Gibbs free energy of the photogenerated electron–hole pair and separated electron and hole are very close. These results reveal that OSCs are thus not limited by the Coulomb energies that bind excitons and CTEs, due to the possibility of undergoing thermally activated charge separation. We note that recent work by Perdigón-Toro et al.^50^ shows high charge photogeneration quantum yields at cryogenic temperatures in an efficient non-fullerene OSC system (PM6:Y6), thus suggesting that the separation of CT states is barrierless. We consider that this result is observed even if the CT states need to overcome a significant energy barrier to separate, provided that the charges are sufficiently long-lived such that they can separate by thermal activation even at reduced temperatures (though at a reduced rate).

Finally, future OSCs should be designed to remove all irreversible processes to further reduce non-radiative recombination losses. This could be achieved for example via the use of donor and acceptor materials with slow vibrational relaxation and high PL quantum yields such that regeneration of excitons via free electron–hole encounters leads to either: (1) redissociation into CTEs and subsequently into free carriers or (2) radiative recombination. It is also now time to address issues such as quenching of PL at charge collection electrodes, much investigated for lead halide perovskite PV systems, that have hitherto not been regarded as critical to performance for OSCs. This will enable OSCs to achieve high charge generation efficiencies with small photovoltage losses that are comparable to non-excitonic solar cells based on inorganic and hybrid semiconductors.

## Methods

### Materials and sample preparation

P3TEA, P3TAE, PffBT2T-TT, SF-PDI_2_, FTTB-PDI_4_, and O-IDTBR were synthesized and provided by Prof. He Yan, and PCBM was purchased from 1-Materials. P3TEA:SF-PDI_2_ and P3TEA:PCBM (both 1:1.5 w/w, polymer concentration 9 mg ml^−1^) blends and pristine P3TEA films were prepared in 1,2,4-trimethylbenzene (TMB) with 2.5% of 1,8-octanedithiol (ODT) and left for stirring at 100 °C for at least 1 h prior to film deposition. Quartz substrates were cleaned by sonicating in deionized water, acetone, and isopropanol for 5 min successively, followed by oxygen plasma treatment for 10 min. Thin-films were spin-coated from hot solutions on the preheated substrate in a N_2_ filled glove box at 1500 r.p.m. to obtain thicknesses of ~100 nm and then thermally annealed for 10 min. Devices were prepared by spin-coating the photoactive materials onto precleaned ITO-coated glass instead of quartz substrates. Then, a thin layer (20 nm) of V_2_O_5_ was deposited as the anode interlayer, followed by deposition of 100 nm of Al as the top electrode (at a vacuum level of 3 × 10^−6^ torr). All thin-film and device samples were encapsulated using epoxy and thin glass slides. Each device pixel had an area of 5.9 mm^2^, which is defined by a metal mask with an aperture aligned with the device area.

### Steady-state optical and electrical measurements

Ultraviolet–visible absorption spectra of thin-film samples were acquired using a Perkin Elmer Lambda 20 UV/VIS Spectrophotometer. Device *J*–*V* characteristics were measured under AM 1.5 G (100 mW cm^−2^) at room temperature using a Newport solar simulator. The light intensity was calibrated using a standard Si diode (with KG5 filter, purchased from PV measurement) to bring spectral mismatch to unity. EQE spectrum was characterized with a Newport EQE system with a standard Si diode. The quasi-steady-state EA spectrum was acquired by measuring the relative change in reflection off the back electrode (∆*R*/*R*) at various wavelengths in response to a varying electric field applied across the electrodes of the device, with the beam passing through the donor–acceptor blend twice. The device was held under a small (−1 V) reverse bias to minimize charge injection from the electrodes. Since this small bias does not affect the reflectance of the back electrode, the relative change in reflection is equivalent to the relative change of transmission (∆*T*/*T*) through the sample. GIWAXS measurements were carried out on a Xenocs Xeuss 2.0 system with an Excillum MetalJet-D2 X-ray source operated at 70.0 kV, 2.8570 mA, and a wavelength of 1.341 Å. The grazing-incidence angle was set at 0.20°. Scattering pattern was collected with a ﻿Pilatus 1 M two-dimensional detector. The time-integrated PL and electroluminescence (EL) spectra of thin-film and device samples were measured using a calibrated Andor iDus CCD camera. An integrating sphere was used to determine PL quantum yields. The device samples were connected to a Keithley 2400 source meter for studying the change in PL at reverse bias and for driving EL at forward bias.

### PP transient absorption spectroscopy

A Ti:sapphire amplifier (Coherent Legend Elite 1 kHz, 150 fs, 800 nm) seeded by a Ti:sapphire oscillator (Coherent Mira 900) was used to provide output pulses about 4 mJ. The output of amplifier was split into two optical beams. One beam was used to generate the pump beam using an optical parametric amplifier (OPA) system (Light Conversion OPerA SOLO 200–2500 nm). The other beam was focused onto a sapphire crystal to generate white-light continuum as the probe beam at 1 kHz (a notch filter is used to cut the fundamental 800 nm component). An optical delay stage was used to add a time delay between the pump and probe pulses. A chopper wheel was used to chop the pump beam at 500 Hz, and provide the synchronization for the detector. The power of pump beam was attenuated with a N.D. filter to get a fluence about 1.5 μJ cm^−2^ per pulse. Pump and probe beams were spatially overlapped on the sample and the transmitted probe light was collected by a spectrometer (Acton SpectraPro 275) equipped with a Si CCD camera (Entwicklungsbuero Stresing CCD 2000). The change in transmission (∆*T*/*T*) was detected at various time delays. For low temperature measurements, the samples were mounted in a liquid helium continuous flow cryostat, and the sample temperature was controlled via an active feedback heater and temperature controller. An electronically delayed Q-switched Nd:YVO4 laser (532 nm, Advanced Optical Technologies Ltd.) was used to provide pump pulses for measurements beyond 1 ns.

### PPP transient absorption spectroscopy

All optical pulses were generated from a PHAROS (Light Conversion) laser running at 38 kHz. The probe pulse was a chirped white-light continuum generated by focusing the fundamental (1030 nm) in a YAG crystal. The 670 nm pump pulse was generated in a home-built non-collinear OPA, and the 800 nm push pulse was generated by feeding the PHAROS output into a commercial ORPHEUS (Light Conversion) OPA. The optical pulses were spatially overlapped in the sample through a boxcar geometry, and temporally delayed using a piezoelectric delay stage for the PP delay and a DC servo stage for the pump–push delay. After passing the sample, the probe was split with a low pass filter with 960 nm cut-off to obtain a transmitted near-infrared beam and a reflected visible beam. The near infrared and visible probe beams were guided into a spectrometer with an InGaAs and Si photodiode array detector, respectively. The two sensors were synchronized and detected simultaneously at 38 kHz, enabling shot-to-shot detection. Pump and push beams were chopped at 38/4 and 38/8 kHz, respectively, such that we simultaneously recorded the pump-induced change in transmission (∆*T*/*T*) and the pump–push-induced response (∆(∆*T*/*T*)). The push-induced change in transmission in the absence of a pump (push–probe response) was also recorded, which allowed us to minimize multi-photon excitations of the ground state by the push. The pump and push fluences were set to obtain a good signal to noise for the PPP signal ∆(∆*T*/*T*) with minimum pump and push fluences, and the PP signal measured was used to check for sample degradation.

### Time-resolved PL spectroscopy

The encapsulated thin-film or device samples were photoexcited with a Fianium laser running at 5 MHz. A 680 nm band-pass filter was used to select the excitation wavelength. The collected PL was measured with an InGaAs time-correlated single photon counter. Long-pass and band-pass filters were used for selecting specific detection ranges. A Keithley 2400 source meter was used to study the change in PL decay at various bias conditions for devices.

## Supplementary information

Supplementary Information

## Data Availability

The data supporting the results of this work are available from the corresponding authors upon reasonable request.

## References

[1] Coropceanu V, Chen X-K, Wang T, Zheng Z, Brédas J-L (2019). Charge-transfer electronic states in organic solar cells. Nat. Rev. Mater..

[2] Gelinas S (2014). Ultrafast long-range charge separation in organic semiconductor photovoltaic diodes. Science.

[3] Shockley W, Queisser HJ (1961). Detailed balance limit of efficiency of p-n junction solar cells. J. Appl. Phys..

[4] Benduhn J (2017). Intrinsic non-radiative voltage losses in fullerene-based organic solar cells. Nat. Energy.

[5] Rao A (2013). The role of spin in the kinetic control of recombination in organic photovoltaics. Nature.

[6] Chow PCY, Gélinas S, Rao A, Friend RH (2014). Quantitative bimolecular recombination in organic photovoltaics through triplet exciton formation. J. Am. Chem. Soc..

[7] Rau U (2007). Reciprocity relation between photovoltaic quantum efficiency and electroluminescent emission of solar cells. Phys. Rev. B.

[8] Causa’ M (2016). The fate of electron–hole pairs in polymer:fullerene blends for organic photovoltaics. Nat. Commun..

[9] Provencher F (2014). Direct observation of ultrafast long-range charge separation at polymer–fullerene heterojunctions. Nat. Commun..

[10] Tvingstedt K (2009). Electroluminescence from charge transfer states in polymer solar cells. J. Am. Chem. Soc..

[11] Menke SM (2018). Order enables efficient electron-hole separation at an organic heterojunction with a small energy loss. Nat. Commun..

[12] Ran NA (2016). Harvesting the full potential of photons with organic solar cells. Adv. Mater..

[13] Kawashima K, Tamai Y, Ohkita H, Osaka I, Takimiya K (2015). High-efficiency polymer solar cells with small photon energy loss. Nat. Commun..

[14] Ullbrich S (2019). Emissive and charge-generating donor–acceptor interfaces for organic optoelectronics with low voltage losses. Nat. Mater..

[15] Vandewal K (2012). Quantification of quantum efficiency and energy losses in low bandgap polymer:fullerene solar cells with high open-circuit voltage. Adv. Funct. Mater..

[16] Li W, Hendriks KH, Furlan A, Wienk MM, Janssen RAJ (2015). High quantum efficiencies in polymer solar cells at energy losses below 0.6 eV. J. Am. Chem. Soc..

[17] Dimitrov SD (2012). On the energetic dependence of charge separation in low-band-gap polymer/fullerene blends. J. Am. Chem. Soc..

[18] Vandewal K, Tvingstedt K, Gadisa A, Inganäs O, Manca JV (2010). Relating the open-circuit voltage to interface molecular properties of donor:acceptor bulk heterojunction solar cells. Phys. Rev. B.

[19] Yao J (2015). Quantifying losses in open-circuit voltage in solution-processable solar cells. Phys. Rev. Appl..

[20] Wang Y (2018). Optical gaps of organic solar cells as a reference for comparing voltage losses. Adv. Energy Mater..

[21] Baran D (2018). Robust nonfullerene solar cells approaching unity external quantum efficiency enabled by suppression of geminate recombination. Nat. Commun..

[22] Chen S (2017). A wide-bandgap donor polymer for highly efficient non-fullerene organic solar cells with a small voltage loss. J. Am. Chem. Soc..

[23] Li S (2018). A wide band gap polymer with a deep highest occupied molecular orbital level enables 14.2% efficiency in polymer solar cells. J. Am. Chem. Soc..

[24] Liu J (2016). Fast charge separation in a non-fullerene organic solar cell with a small driving force. Nat. Energy.

[25] Nikolis VC (2017). Reducing voltage losses in cascade organic solar cells while maintaining high external quantum efficiencies. Adv. Energy Mater..

[26] Zhang G (2018). Nonfullerene acceptor molecules for bulk heterojunction organic solar cells. Chem. Rev..

[27] Yuan J (2019). Single-junction organic solar cell with over 15% efficiency using fused-ring acceptor with electron-deficient core. Joule.

[28] Cui Y (2020). Single-junction organic photovoltaic cells with approaching 18% efficiency. Adv. Mater..

[29] Qian D (2018). Design rules for minimizing voltage losses in high-efficiency organic solar cells. Nat. Mater..

[30] Chen XK, Coropceanu V, Brédas JL (2018). Assessing the nature of the charge-transfer electronic states in organic solar cells. Nat. Commun..

[31] Eisner FD (2019). Hybridization of local exciton and charge-transfer states reduces nonradiative voltage losses in organic solar cells. J. Am. Chem. Soc..

[32] Classen A (2020). The role of exciton lifetime for charge generation in organic solar cells at negligible energy-level offsets. Nat. Energy.

[33] Panhans M (2020). Molecular vibrations reduce the maximum achievable photovoltage in organic solar cells. Nat. Commun..

[34] Schwarze M (2019). Impact of molecular quadrupole moments on the energy levels at organic heterojunctions. Nat. Commun..

[35] Yao H (2019). 14.7% Efficiency organic photovoltaic cells enabled by active materials with a large electrostatic potential difference. J. Am. Chem. Soc..

[36] Zhang G (2020). Delocalization of exciton and electron wavefunction in non-fullerene acceptor molecules enables efficient organic solar cells. Nat. Commun..

[37] Zhang J (2017). Ring-fusion of perylene diimide acceptor enabling efficient nonfullerene organic solar cells with a small voltage loss. J. Am. Chem. Soc..

[38] Liu J (2018). Carboxylate substitution position influencing polymer properties and enabling non-fullerene organic solar cells with high open circuit voltage and low voltage loss. J. Mater. Chem. A.

[39] Chen S (2018). Efficient nonfullerene organic solar cells with small driving forces for both hole and electron transfer. Adv. Mater..

[40] Zhong Y (2020). Sub-picosecond charge-transfer at near-zero driving force in polymer:non-fullerene acceptor blends and bilayers. Nat. Commun..

[41] Wang R (2019). Ultrafast hole transfer mediated by polaron pairs in all-polymer photovoltaic blends. Nat. Commun..

[42] Wang R (2020). Charge separation from an intra-moiety intermediate state in the high-performance PM6:Y6 organic photovoltaic blend. J. Am. Chem. Soc..

[43] Vandewal K (2014). Efficient charge generation by relaxed charge-transfer states at organic interfaces. Nat. Mater..

[44] Burke TM, Sweetnam S, Vandewal K, McGehee MD (2015). Beyond Langevin recombination: how equilibrium between free carriers and charge transfer states determines the open-circuit voltage of organic solar Cells. Adv. Energy Mater..

[45] Gao F, Tress W, Wang J, Inganäs O (2015). Temperature dependence of charge carrier generation in organic photovoltaics. Phys. Rev. Lett..

[46] Jakowetz AC (2017). Visualizing excitations at buried heterojunctions in organic semiconductor blends. Nat. Mater..

[47] Chow PCY, Bayliss SL, Lakhwani G, Greenham NC, Friend RH (2015). In situ optical measurement of charge transport dynamics in organic photovoltaics. Nano Lett..

[48] Koster LJA, Kemerink M, Wienk MM, Maturová K, Janssen RAJ (2011). Quantifying bimolecular recombination losses in organic bulk heterojunction solar cells. Adv. Mater..

[49] Benduhn J (2018). Impact of triplet excited states on the open-circuit voltage of organic solar cells. Adv. Energy Mater..

[50] Perdigón-Toro, L. et al. Barrierless free charge generation in the high-performance PM6:Y6 bulk heterojunction non-fullerene solar cell. *Adv. Mater*. **32**, 1906763 (2020). 10.1002/adma.201906763 31975446

