# Supplementary material for: Long-lived and disorder-free charge transfer states enable endothermic charge separation in efficient non-fullerene organic solar cells
Source: Nat Commun. 2020 Nov 5;11:5617. doi: 10.1038/s41467-020-19332-5 (PMC7645751; doi:10.1038/s41467-020-19332-5)
Supplement: Supplementary file 1 — Supplementary Information [file 41467_2020_19332_MOESM1_ESM.pdf]

Supplementary Information

Long-lived and disorder-free charge transfer states enable endothermic charge separation in efficient non-fullerene organic solar cells

Ture F. Hinrichsen[1,#], Christopher C.S. Chan[2,#], Chao Ma[2,#], David Paleček[1], Alexander Gillett[1], Shangshang Chen[3], Xinhui Zou[3], Guichuan Zhang[4], Hin-Lap Yip[4], Kam Sing Wong[2], Richard H. Friend[1,*], He Yan[3,*], Akshay Rao[1,*], Philip C.Y. Chow[3,5,*]

1. Cavendish Laboratory, University of Cambridge, Cambridge, CB3 0HE, United Kingdom
2. Department of Physics, The Hong Kong University of Science and Technology, Clear Water Bay, Hong Kong
3. Department of Chemistry, The Hong Kong University of Science and Technology, Clear Water Bay, Hong Kong
4. Institute of Polymer Optoelectronic Materials and Devices, State Key Laboratory of Luminescent Materials and Devices, South China University of Technology, Guangzhou 510640, China
5. Department of Mechanical Engineering, The University of Hong Kong, Pokfulam, Hong Kong

# These authors contributed equally to this work.

Correspondence: PCYC <pcyc@hku.hk>, AR <ar525@cam.ac.uk>, HY <hyan@ust.hk>, or RHF <rhf10@cam.ac.uk>

Supplementary Notes:

- Supplementary Note 1: Additional material and device characterizations
- Supplementary Note 2: Extended technical descriptions of electroabsorption
- Supplementary Note 3: Extended technical descriptions of pump-probe (PP) data
- Supplementary Note 4: Extended technical descriptions of pump-push-probe (PPP) data

Supplementary figures:

- Supplementary Fig. 1-14

Supplementary Note 1: Additional material and device characterizations

1.1. UV-Vis absorption and photovoltaic performance

Supplementary Fig. 2a,b show the absorption profile and energy levels of the materials involved in this study. The absorption data was measured in thin-films and the energy levels were estimated from cyclic voltammetry. Supplementary Fig. 2c,d show the solar cell device performance of the five studied blends. The peak internal quantum efficiency (IQE) is above 85% for the model P3TEA:SF-PDI_2 device. Also summarised is the total voltage loss of each device, which is given by the difference between optical bandgap (E_{gap}/q) and the open-circuit voltage (V_{OC}). All non-fullerene systems studied herein exhibit much lower voltage losses compared to fullerene devices (typically ~0.8-1.0 V). We note that the value of voltage loss depends on how the optical gap is determined (see Wang et al. Adv. Energy Mater. 8, 1801352 (2018)). Here, all optical gaps were determined from the crossing point between absorption and emission spectra, as opposed to the onset of either absorption or emission spectra. We note that even smaller voltage losses (by about ~0.05 V) are calculated for each system using optical gaps taken from the absorption onset, and therefore the values quoted in the table below represent the upper bound of the voltage losses.

1.2. Morphology characterization

Detailed analysis of the blend morphology of P3TEA:SF-PDI_2, P3TAE:SF-PDI_2, PffBT2T-TT-O-IDTBR and P3TEA:FTTB-PDI_4 blends are described in Ref. [1–4]. Using resonant soft-X-ray scattering (R-SOXS) and grazing-incidence wide-angle X-ray scattering (GIWAXS), it was found that all of these blends have similarly small domain sizes (~20-30nm) with high domain purity. Furthermore, by tracking the high-order lamellar stacking peaks, it was found that the donor polymers in these blends exhibit high crystallinity with preferential packing in the out-of-plane direction. Additionally, atomic force microscopy (AFM) measurements taken on these blended films show a uniform and smooth surface without clear phase separation (Supplementary Fig. 3a). The morphologies of these blends are therefore very similar, showing a combination of fine intermixing between donor and acceptor materials and highly pure and crystalline domains which is well-known to be desirable for bulk-heterojunction organic solar cells. The similarity of morphologies in these blends is consistent with their similar charge generation dynamics that we reveal in this work. For P3TEA:SF-PDI_2 and P3TEA:PCBM, our GIWAXS data also show similar polymer packing in the out-of-plane direction (1.75 $Å^{-1}$), thus indicating that they have similar morphology as well (Supplementary Fig. 3b). Further evidence for this is the significant PL quenching shown in both blends (Fig. 4), which implies that both blends have nanoscale domain allowing excitons to diffuse to a heterojunction and separate. These two blends also have similar energetic disorder at the D/A interface (see Section 1.3 for details).

1.3. Sub-gap absorption and emission spectroscopy

We used photo-thermal deflection spectroscopy (PDS) and electroluminescence (EL) spectroscopy to study sub-gap absorption and emission characteristics of P3TEA, P3TEA:SF-PDI_2 and P3TEA:PCBM. Supplementary Fig. 4a shows the PDS data measured in thin films. We find that the three films show similar sub-gap absorption profiles, indicating that charge-transfer states

formed at the donor-acceptor heterojunctions for the two blends have similar energies as the lowest singlet excitons. The steep absorption edges show Urbach energies of ~27 meV, as indicated by the dashed line. This value is considerably smaller than those previously measured for other organic heterojunctions, particularly those involving fullerene acceptors (typically ~40-50 meV). This provides evidence that the energy landscape at the donor-acceptor interface of these blends is relatively ordered and free of deep trap sites that generally lead to fast non-radiative recombination (see Ref. [5]).

Supplementary Fig. 4b,c show the electroluminescence (EL) spectrum of the non-fullerene and fullerene blend, respectively. The coloured lines represent different injection biases (between 1.5 to 3V). We find almost no difference in the emission spectral shape with increasing injection bias for the non-fullerene blend, while for the fullerene blend we find decreasing emission below ~1.5 eV with respect to the peak emission at ~1.65 eV. As described previously (Ref. [6]), this spectral effect is observed in cases where the charge-transfer exciton states (CTEs) have lower energy compared to the singlet excitons. More charges recombine via the CTEs at small injection bias, while more excitons are populated at large injection bias. Therefore, our results indicate that for the non-fullerene blend the CTEs have close energy alignment with the singlet excitons, while for the fullerene blend the CTEs have lower energy than the singlets.

We quantify the CTE energy (E_{CT}) by simultaneously fitting the absorption edge and EL spectra using equations derived from Marcus theory (more details in Ref. [6]), and from that extract the band gap of the emitting state:

$$Abs_{pv}(E) = \frac{f}{E\sqrt{4\pi\lambda kT}} exp\left(\frac{-(E_{CT}+\lambda-E)^2}{4\lambda kT}\right) \qquad (1)$$

$$EL_{pv}(E) = \frac{f}{E\sqrt{4\pi\lambda kT}} exp\left(\frac{-(E_{CT}-\lambda-E)^2}{4\lambda kT}\right) \qquad (2)$$

where E is the photon energy, k is the Boltzmann constant, and T is the temperature. The fit parameters are E_{CT} , the reorganization energy λ , and a factor f proportional to the number of states. The fits are shown as the dashed lines. For P3TEA:SF-PDI_2, we fit the absorption and EL spectra at the peak since we observed no injection bias dependence on the EL spectrum, thus indicating CTEs have very similar energy as the lowest singlet excitons at 1.72 eV. For P3TEA:PCBM, our bias-dependent EL data indicates that CTEs have slightly lower energy than singlets, and their emission is red-shifted towards ~1.5 eV. As described by Ran et al. [6], it is possible to fit the low energy shoulder of the EL as the CTE shoulder. We therefore fit the low energy shoulder of the EL and we estimate an E_{CT} of 1.62 eV for the fullerene blend. Similar data for P3TAE:SF-PDI_2, P3TEA:FTTB-PDI_4 and PffBT2T-TT:O-IDTBR are found in Ref. [2–4].

1.4. Temperature dependent photocurrent measurements

We measured the temperature-dependent photocurrent of P3TEA:SF-PDI_2 and P3TEA:PCBM devices. The device was placed inside a nitrogen cryostat and photoexcited using a 633nm diode laser. Supplementary Fig. 5 shows the short-circuit photocurrent at various temperatures. We find that the photocurrent of P3TEA:SF-PDI_2 device drops more significantly than P3TEA:PCBM, with almost no photocurrent measured at ~100K. This is consistent with the temperature

dependence found for the spectroscopy data as described in the main text. Data for annealed P3HT:PCBM, MEH-PPV:PCBM, PTB7:PCBM, and Si (reproduced from Gao et al. Phys. Rev. Lett. **114,** (2015), Gommans et al. Appl. Phys. Lett. **87,** 1–3 (2005), Ebenhoch et al. Org. Electron. **22,** 62–68 (2015), and Singh & Ravindra, Sol. Energy Mater. Sol. Cells 101, 36 (2012), respectively) are shown for comparison. We note that all device photocurrents were measured at sufficiently low intensity to avoid a current drop due to bimolecular recombination (with the exception of PTB7:PCBM which was measured under simulated solar illumination).

1.5. Additional time-resolved photoluminescence spectroscopy

Supplementary Fig. 6 shows time-resolved PL of P3TEA:SF-PDI$_2$ at various detection wavelengths. As discussed in the main text, this delayed emission is observed only for the non-fullerene blend and not for the pure polymer or fullerene blended films (see Fig. 4) and is attributed to regeneration of emissive singlet excitons or CTEs upon recombination events of free carriers. We find little difference in the PL decay at various detection wavelengths, thus confirming that the exciton and CTE states are indeed very similar in energy (therefore allowing reversible interconversions between these states).

Supplementary Note 2: Extended technical descriptions of electroabsorption

In this section we will explain the origin of the electroabsorption (EA) signals that we used to study charge separation in greater detail. The EA signal is created by a Stark effect of the absorption spectrum of the organic semiconductor. The Stark effect can be thought of as the shifting of a localized state energy in the presence of an electric field (E). The change in the excited state energy, ΔU is given by (10.1021/acs.jpclett.7b01741):

$$\Delta U(E) \propto -\Delta\mu E - \frac{1}{2}\Delta p E^2 \qquad (3)$$

where Δμ is the change in dipole moment and Δp is the change in polarizability.

There are 3 types of EA signals described in our study, as illustrated in Supplementary Fig. 7b-d below. Although they have different origins, they are all caused by a uniform Stark shift of the absorption spectrum and therefore have a first-derivative lineshape. The first EA signal is the quasi-steady state EA signal measured in a diode structure. As discussed in the main text, this signal is used as a reference for comparison with the transient EA signals that we observed in pump-probe (PP) and pump-push-probe (PPP) experiments. The key difference between the quasi-steady state EA signal and the other two transient EA signals is that it is created by the macroscopic electric fields induced by the applied bias across the two electrodes, while the others are caused by local electric fields between photoexcitations during charge separation (with/without push excitation).

The second EA signal is observed in pump-probe transient absorption (in $\Delta T/T$). As described in the main text and in previous reports (see Ref. [7]) due to the derivative spectral shape of the EA response, the rise of EA signal causes a spectral blue-shift near the optical gap that provides a

spectral signature for charge separation. It is important to emphasize that, unlike EA measurements performed on diodes, the resulted EA response is induced by multiple randomly oriented ‘dipolar’ fields localized in the vicinity of each photogenerated electron-hole pair. Thus, although the total EA intensity is still $\propto \int |E|^2\, dV$, its origin is completely different. In the macroscopic case, the average electron-hole distance can be calculated assuming a simple model where two sheets of charges of opposite sign move away from one-another due to an applied bias. However, this macroscopic model is not valid for localised dipole-like fields where there is no preferential directionality to charge separation in a bulk heterojunction structure (the donor-acceptor interfaces are randomly oriented and the mean macroscopic field is zero). As described in previous work (see Ref. [7]), this local EA signal can be modelled by a classical electrostatic representation of the microscopic charge separation process where the work done to move an electron to a distance r from the hole is proportional to -1/r (with the energy at infinite distance normalized to zero). As the electron-hole separation increases, the total energy stored in the electric field will tend asymptotically towards that of two independent charges. Therefore, the change in electrostatic energy induced by moving these charges further apart becomes small once the electron-hole pair are ‘free’ from their mutual attraction. The electro-absorption induced by a charge pair will therefore follow the same trend. This implies that the local EA response is specifically sensitive to the separation from bound to free charges (signal saturates at ~5 nm separation). This signal is thus a signature for the charge separation process. As discussed in the main text, this signal is observed for both fullerene and non-fullerene blends, consistent with the high photocurrent IQE measured for both devices, but arising at very different rates (~100 fs vs. ~100 ps) and with contrasting temperature dependence.

The third EA signal is observed in pump-push-probe transient absorption (in $\Delta(\Delta T/T)$). As we discussed in main text, this signal arises upon push-excitation of bound charge-transfer excitons (CTEs) which stay localised at the donor-acceptor heterojunction. Absorption of the push energy allows the interfacial electron-hole pairs to spatially separate across the heterojunction, creating a local field that results in a microscopic EA response as described above. It is important to note that this push-induced EA signal is only observed in the presence of CTEs (as in the non-fullerene blends), and is not observed either when we push excitons (as in pristine film) or free carriers (as in the fullerene blends where most photoexcitations quickly separate into free charges). Therefore, the push-induced EA signal provides a direct probe of the localised CTE population at the donor-acceptor interface.

It is known that an EA signal caused by a Stark effect can have a first or second derivative lineshape (see Bublitz and Boxer, 10.1146/annurev.physchem.48.1.213). In cases where the Stark effect induces a change of polarizability of a transition, the electric field interaction will create a dipole moment only in the direction of the field, regardless of the molecule’s orientation. As a result the entire absorption spectrum shifts uniformly to a lower energy, leading to an EA spectrum in the form of the first derivative of the absorption spectrum.

Supplementary Note 3: Extended technical descriptions of pump-probe (PP) data

3.1. Non-normalised transient absorption data and spectral analysis

Supplementary Fig. 8a-c shows the non-normalised transient absorption (TA) data of pure P3TEA, P3TEA:SF-PDI_2 and P3TEA:PCBM thin-films at room temperature, photoexcited at 670 nm at fluence of 1.5 µJ cm^{-2} per pulse. The normalised data is shown in Fig. 2 in main text. These TA results were taken using a white-light continuum (~550-800 nm) created in a visible NOPA using the output of a Ti-Sapphire laser (see Methods). This wavelength window is ideal for studying spectral evolution close to the optical gap of P3TEA systems (~700-750 nm). Detailed description of the TA spectral evolution is found in the main text. We note that, since the selected excitation energy is well below the absorption edge of SF-PDI_2 and PCBM, we preferentially create photoexcitations in the P3TEA donor polymers. Under the same excitation condition, we observe no TA signal above the noise limit for a pure acceptor sample. We do observe a TA response when we directly excite the acceptor material at 500 nm. However, the intensity of the resulted TA signal is very weak ($<10^{-5}$). As shown in Supplementary Fig. 8d, the TA response of the pure SF-PDI_2 film is only $\sim 2\times10^{-3}$ when excited at a fluence of ~30 µJ cm^{-2} per pulse. Under the same excitation fluence and accounting for difference in absorbance, we obtain a peak TA response of $\sim 2\times10^{-2}$ for P3TEA and the blended films. This indicates that the absorption cross-section of excited states (both excitons and polarons) in the acceptor molecule is about an order of magnitude lower than the donor polymer, and therefore their presence in the blended films do not significantly affect our analysis. This is further confirmed by the largely overlapping TA response of P3TEA:SF-PDI_2 at early times under excitation at 500 nm and 670 nm (see Supplementary Fig. 8e; i.e. the resulted spectra should look very different if excitons and polarons in the acceptor have comparable absorption cross-section to those in the polymer). We note that similarly small TA absorption cross-section was also found for excited states in PCBM (see 10.1002/adma.201400846). For this reason, excited states in the acceptor domain can be neglected in our analysis.

We also measured TA data of the same samples over a wider spectral window (~580-1400 nm) by probing with a white light continuum generated by focusing the fundamental output of a PHAROS laser (1030nm) in a YAG crystal (see Methods). The results are summarized in Supplementary Fig. 9. The results between ~580-780 nm are largely similar to those measured with the other TA setup as shown above. Here we attempt to differentiate the TA response of singlet excitons and polarons in P3TEA polymer. Since excitons generally require a donor-acceptor heterojunction to separate into charges, we consider that the TA response measured in the pure P3TEA film is mostly due to excitons. Although there is no spectral evolution between ~580-780 nm and ~1100-1400 nm, there is slight spectral change between ~780-950 nm for the pure P3TEA film. We note that this spectral region overlaps with the exciton emission spectrum. This spectral change can be due to either: 1) decrease in stimulated emission as excitons move towards less emissive sites, or 2) a small population of excitons that do manage to separate at disordered regions, forming polarons (see Reid, O. G et. al, Charge Photogeneration in Neat Conjugated Polymers. Chem. Mater. 26, 2014). Nevertheless, excitons are expected to be the dominant species in the pure film and therefore the resulting TA response can be attributed mostly to excitons (see blue line in Supplementary Fig. 9d).

As described in the main text, singlet excitons are the dominant species at early times (<2 ps) in P3TEA:SF-PDI_2, while at later times there exists an equilibrium of excitons, CTEs and free carriers. Note that both CTEs and free carriers have polaronic TA signatures. For this reason, it is difficult to isolate the polaron response in the non-fullerene blend. On the other hand, excitons are able to quickly separate into free carriers in P3TEA:PCBM, and charges do not have sufficient energy to regenerate excitons upon recombination. Therefore, the measured TA spectrum at ~2-20 ps for the fullerene blend can be attributed to polarons, with substantial contribution from electroabsorption (EA) that causes spectral blue-shift near the optical edge (see red line in Supplementary Fig. 9d).

3.2. Ultrafast and temperature-independent charge separation in P3TEA:PCBM

Supplementary Fig. 10a shows the TA spectrum of pure P3TEA film at various temperatures (photoexcited at 670 nm at a fluence of ~1.5 μJ cm^{-2}). We observe a small spectral red-shift with reducing temperature, which is attributed to lowering of the optical gap. We observe little change in spectral shape with increasing time delay at all studied temperatures. Supplementary Fig. 10b,c show the TA data of P3TEA:SF-PDI_2 and P3TEA:PCBM films studied at the same conditions. As described in the main text and Fig. 2d, the TA spectrum for the non-fullerene blend largely overlaps with the pure polymer film at early times (before ~2 ps), thus indicating that only excitons are present. Charges are able to form at later time, as evident by the spectral blue-shift caused by a growth in electroabsorption (EA) signal, but only when there is sufficient thermal energy (takes ~100 ps to complete at room temperature). For the fullerene blend, there is clear spectral blue-shift with respect to the pure P3TEA, thus indicates the presence of a significant EA signal even at a very early time scale (~0.2-1ps) and at cryogenic temperature (22K). This result is consistent with previous reports that show ultrafast and temperature-independent charge separation in fullerene blends (Ref. [7,8])

3.3. Triplet recombination at nanosecond timescale in P3TEA:PCBM

Bimolecular electron-hole encounters at the heterojunction will generate thermalized CTEs, which can either have spin-singlet or spin-triplet characteristics according to spin-statistics (Ref. [9,10]). As shown in Supplementary Fig. 11, pump-probe transient absorption measurements show no evidence for triplet exciton formation in P3TEA:SF-PDI_2 on any timescale (top of panel b). In contrast, for the P3TEA:PCBM blend, our results show clear spectral red shift on nanosecond timescales that can be attributed to the formation of long-lived triplet excitons via bimolecular recombination (bottom of panel b). Panel c shows the decay of charges and the concomitant rise of triplets in the fullerene blend with the inset showing the triplet and charge spectra extracted. The triplet response shows overlap between the triplet photoinduced absorption signal and the 0-1 ground state transition at ~600-650nm. We speculate that the close energy alignment of the S_1 and CTE energy level suppresses triplet formation in the non-fullerene system, thereby allowing lower non-radiative recombination losses compared to the fullerene system.

Supplementary Note 4: Extended technical descriptions of pump-push-probe (PPP) data

In this section we will explain the different components of the pump-push-probe (PPP) signal in greater detail. Figure S12a shows the PPP spectra of the P3TEA:SF-PDI_2 blend at pump-push delays from 0.8 to 800 ps. As mentioned in the main text, the signal primarily consists of two components: At long pump-push delays the spectrum is dominated by a derivative-like spectrum that can be attributed to an electroabsorption signal related to bound CTE states. The discussion in the main text is focused on this component, as it provides key insights into the charge separation dynamics investigated here. Here we describe the origin of the other component, which dominates at short pump-push delays.

Figure S12b compares this PPP signal at short push-probe delays with the inverted and scaled pump-probe (PP) signal measured at the same pump-probe delay. They match very well, which can be explained as follows: The push reduces the intensity of the transient absorption signal without significant changes in the spectrum. When calculating the difference in absorption, $\Delta\big(\Delta(T/T)\big) = \big(\Delta(T/T)\big)_{push} - \big(\Delta(T/T)\big)_{no\ push}$, this results in a signal that closely resembles the inverted PP spectrum. This reduction in Δ(T/T) signal indicates an overall reduction in excited state population. This is likely due to increased exciton-exciton annihilation of high-energy excitons formed by absorbing the push pulse (see Martini et al, Phys. Rev. B 2004, 69 (3), 1–12.), which also reduces the population of any states that form from the initial singlet excitons at later times. Supplementary Fig. 12c shows the effect of temperature on the long-lived PPP signal in P3TEA:SF-PDI_2.

If this signal results from singlet excitons absorbing the push, we expect a similar signal in the pristine P3TEA film where no CTE states are formed. As shown in Supplementary Fig. 13a, this is indeed the case. The spectra at 0.2 ps and 200 ps pump-push delay are similar and no derivative-like signal is present.

Since we know the origin and the shape of the spectrum that results from absorption by singlet excitons, we can remove this component and isolate the CTE response. We achieve this by subtracting the inverted pump-probe signal, scaled to minimise the signal around 900nm. We note that at very long pump-push delays (e.g, 800 ps) there is no contribution from the singlet annihilation signal anymore. Additional PPP results for P3TEA:PCBM are shown in Supplementary Fig. 13b,c, and additional PPP results for P3TEA:FTTB-PDI_4, P3TAE:SF-PDI_2 and PffBT2T-TT:O-IDTBR are shown in Supplementary Fig. 14.

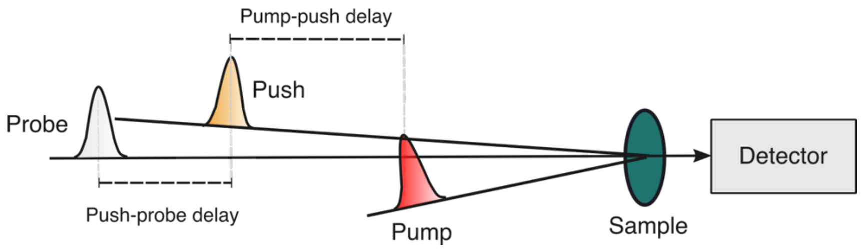

Supplementary Fig. 1. Schematic illustration of time-resolved optical technique used in this work.

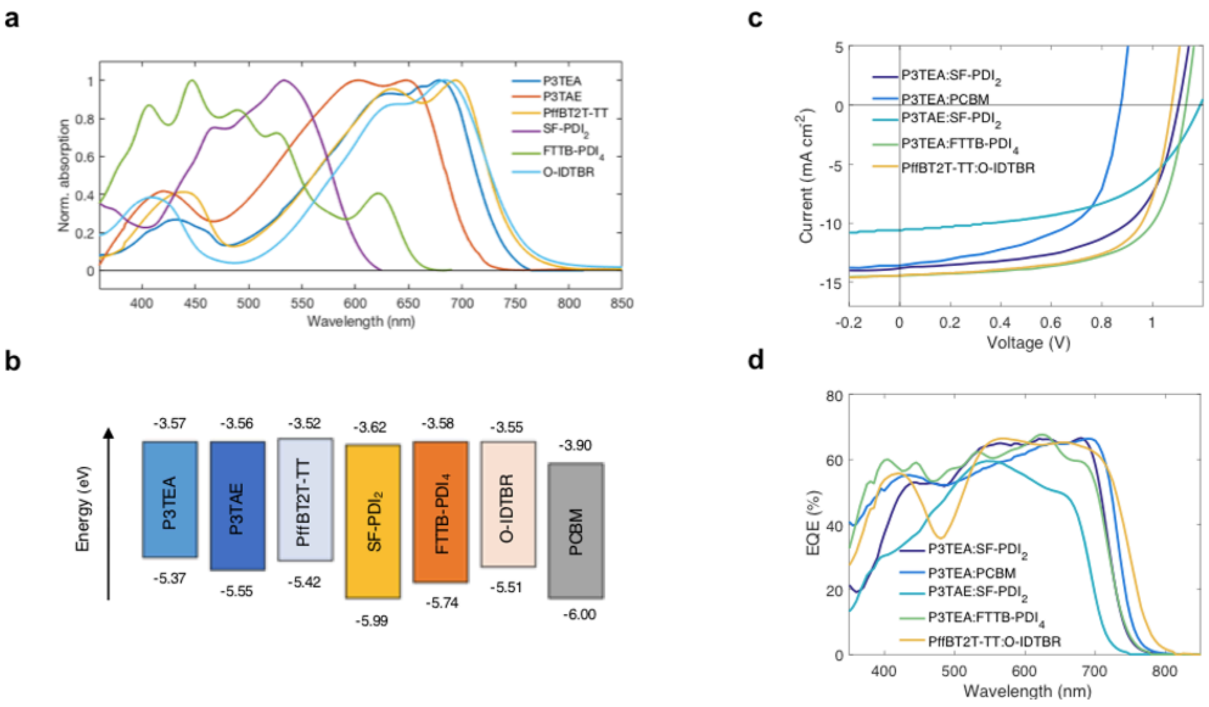

	*E_{gap}/q (V)	V_{oc} (V)	J_{sc} (mA cm^{-2})	FF (%)	PCE (%)	*$E_{gap}/q - V_{oc}$ (V)	EQE_{EL}	IQE (%)
**P3TEA:SF-PDI₂**	1.72	1.11	13.27	64.3	9.5	0.61	5.0×10^{-5}	~85 (Ref. 1)
**P3TEA:PCBM**	1.72	0.89	12.41	69.6	7.5	0.83	1.0×10^{-6}	-
**P3TAE:SF-PDI₂**	1.74	1.19	10.98	55.0	7.12	0.55	4.5×10^{-4}	-
**P3TEA:FTTB-PDI₄**	1.72	1.13	13.89	65.9	10.58	0.59	1.0×10^{-4}	~80 (Ref. 4)
**PffBT2T-TT:O-IDTBR**	1.63	1.08	14.32	67.0	10.4	0.55	1.0×10^{-4}	-

Supplementary Fig. 2. Material and device characterization. (a) Normalised UV-Vis data of materials involved in this study, all measured in thin-films. (b) Energy levels estimated from cyclic voltammetry. (c) Device J-V curves taken under simulated AM 1.5G sunlight. (d) External quantum efficiencies (EQE) at short-circuit condition. Device parameters are summarised in the table. *Optical gaps were determined from crossing of absorption/emission spectra.

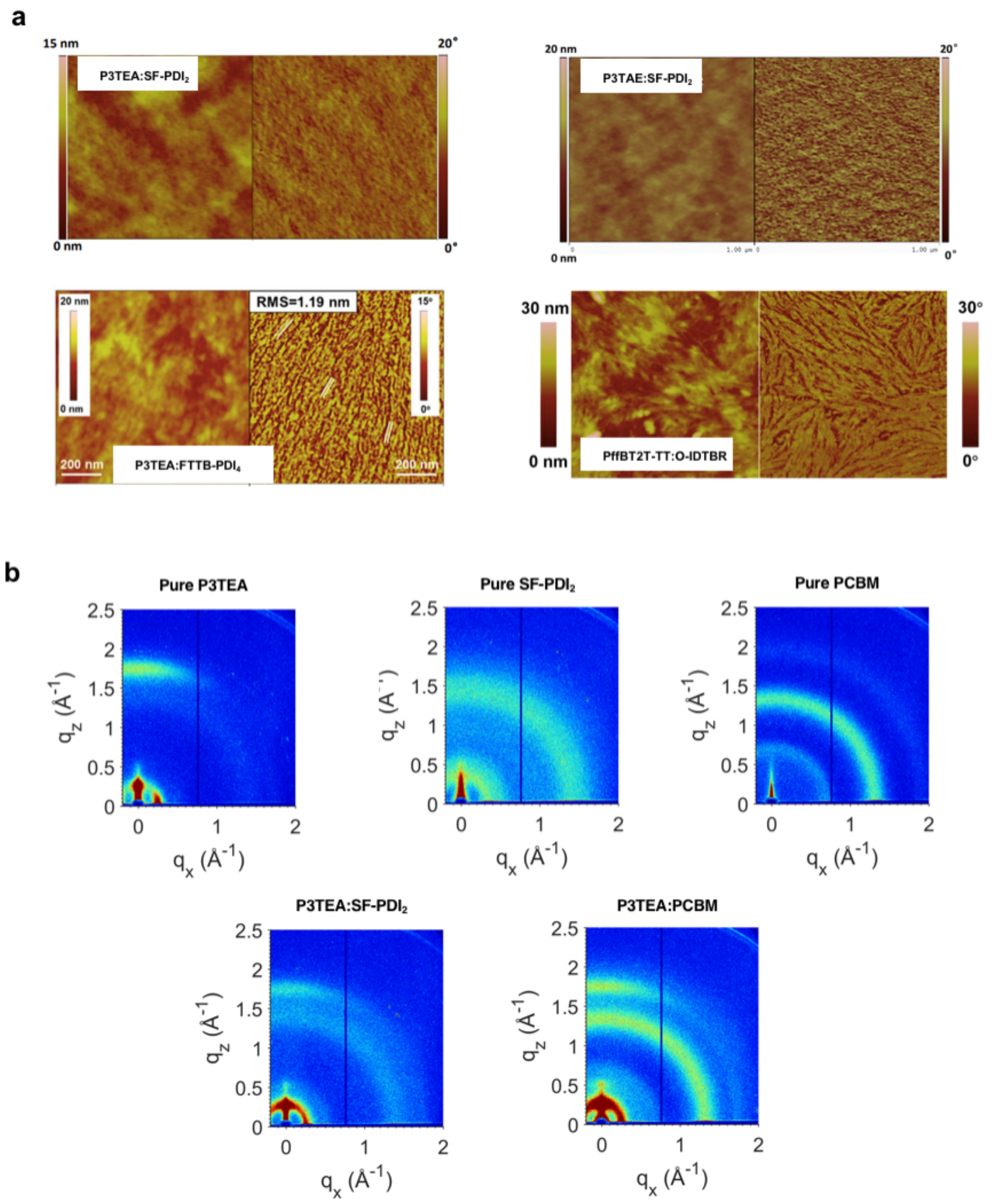

Supplementary Fig. 3. (a) Atomic force microscopy (AFM) data showing uniform and smooth surface without clear phase separation in the non-fullerene blends. (b) Grazing-incidence wide-angle X-ray scattering of pure P3TEA, pure SF-PDI$_2$, pure PCBM, P3TEA:SF-PDI$_2$ and P3TEA:PCBM.

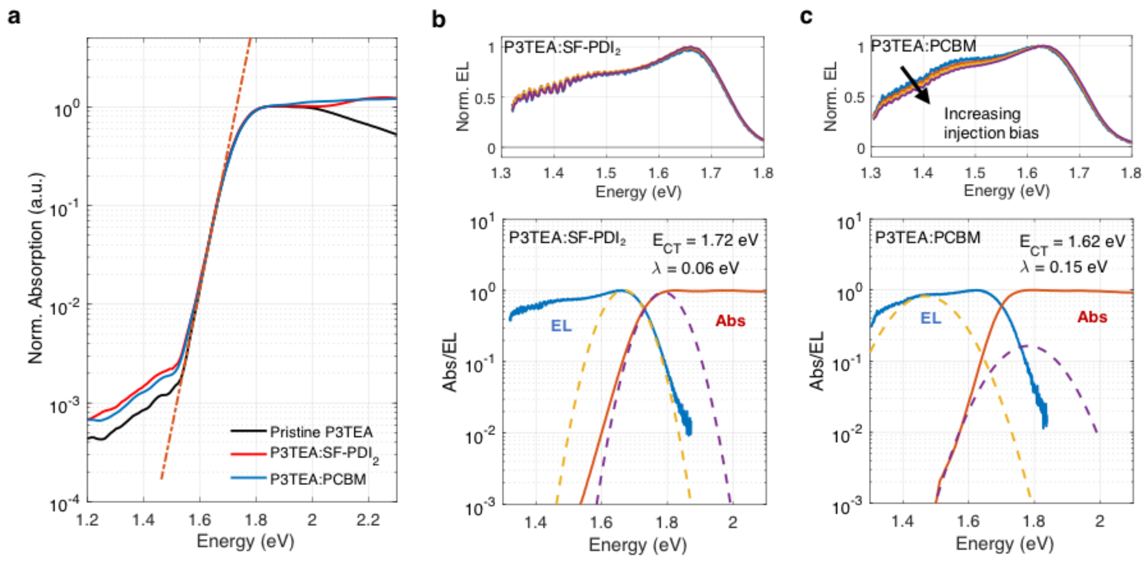

Supplementary Fig. 4. Sub-bandgap absorption and emission. (a) Photo-thermal deflection spectroscopy (PDS) data of pristine P3TEA, P3TEA:SF-PDI$_2$ and P3TEA:PCBM films. The steep absorption edges show small Urbach energies of ~27 meV as indicated by the dashed line. (b,c) Electroluminescence (EL) spectrum of P3TEA:SF-PDI$_2$ (b) and P3TEA:PCBM (c) at various injection biases (between 1.5-3V) and simultaneous fitting of absorption edge and EL spectra with CTE energy (E_{CT}) and renormalization energy (λ) as fitting parameters, as described in Supplementary Note 1.3.

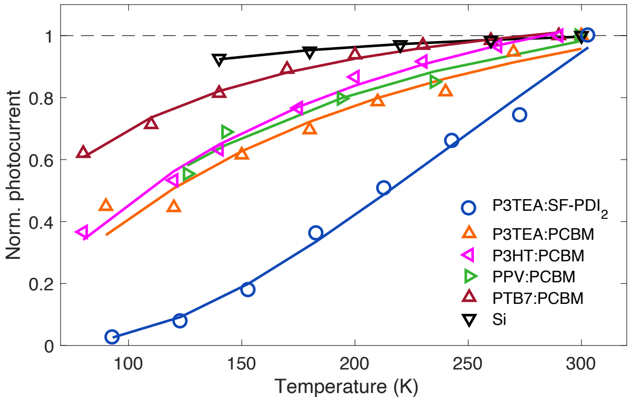

Supplementary Fig. 5. Temperature dependence of device photocurrent. The device was placed inside a nitrogen cryostat and photoexcited using a 633nm diode laser. Data for annealed P3HT:PCBM, MEH-PPV:PCBM, PTB7:PCBM, and Si (reproduced from Gao et al. Phys. Rev. Lett. 114, (2015), Gommans et al. Appl. Phys. Lett. 87, 1–3 (2005), Ebenhoch et al. Org. Electron. 22, 62–68 (2015), and Singh & Ravindra, Sol. Energy Mater. Sol. Cells 101, 36 (2012)) are shown for comparison.

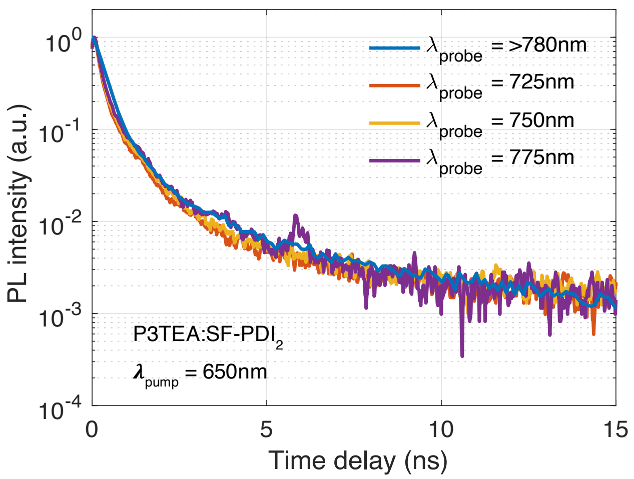

Supplementary Fig. 6. Time-resolved PL decay of P3TEA:SF-PDI$_2$ thin-film photoexcited at 650 nm and probed at various wavelengths, showing negligible difference in decay lifetimes.

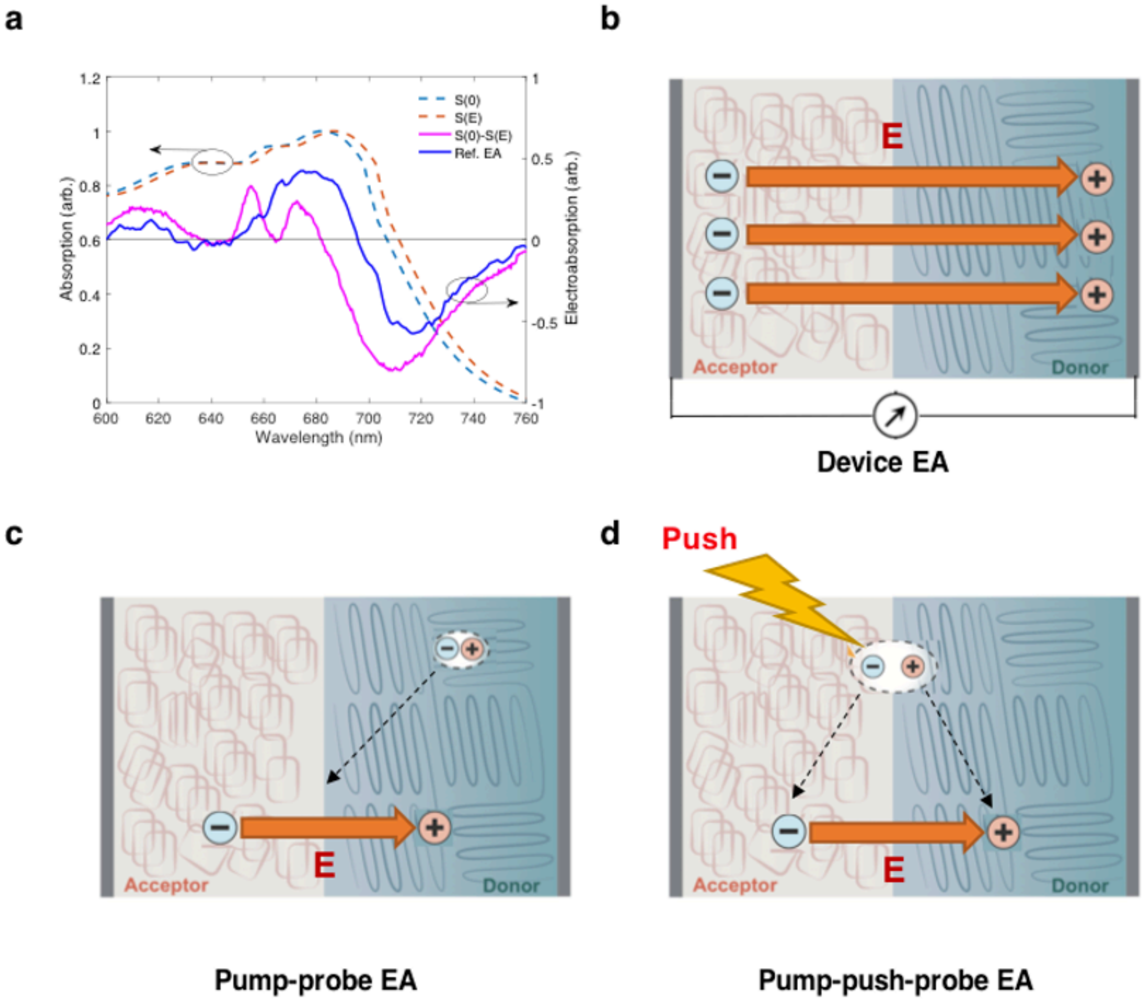

Supplementary Fig. 7. (a) Stark shift of the absorption spectrum (S) due to an electric field E and the resulting electroabsorption response calculated from the difference between the shifted and unshifted spectra. The EA amplitude is proportional to $|E|^2$. The blue line shows the quasi-steady state EA measured in a diode structure for comparison. (b,c,d) Schematic illustrations of the 3 types of EA signals described in this study: EA signal caused by an external electric field (b), EA signal caused by photogenerated charges separating at the interface (c), and EA signal caused by charges initially bound at the interface that separate after interacting with the push pulse (d). Note that only a single donor-acceptor heterojunction is illustrated for simplicity. In reality the donor and acceptor are largely intermixed (bulk-heterojunction) with nanoscale domains, and the heterojunctions are randomly oriented.

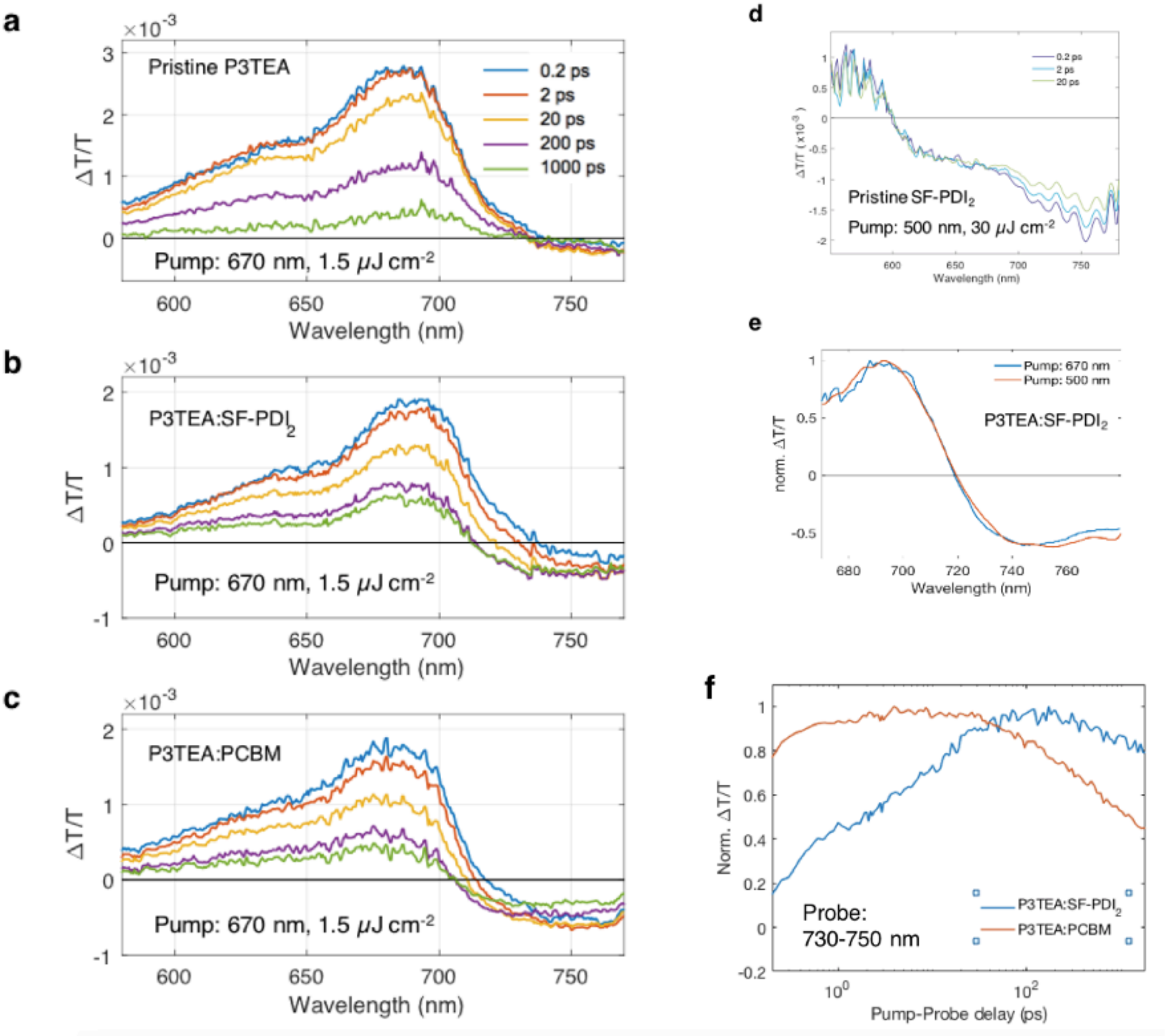

Supplementary Fig. 8. (a,b,c) Un-normalised pump-probe transient absorption (TA) data of pristine P3TEA, P3TEA:SF-PDI$_2$ and P3TEA:PCBM. Normalised plots are shown in Fig. 2 in main text. (d) TA data of pristine SF-PDI$_2$ film, showing much weaker signal compared to samples containing P3TEA under same excitation density. We estimate the absorption cross-section of excited states in the acceptor is about an order of magnitude weaker compared to those in the donor polymer (similar values are also found for PCBM). (e) Normalised TA spectrum of P3TEA:SF-PDI$_2$ under excitation at 500 and 670 nm. The absence of a significant spectral evolution justifies neglecting the excited states in the acceptor domain when analysing the TA spectral evolution at this wavelength window. (f) Normalized TA kinetics probed at 730-750 nm for P3TEA:SF-PDI$_2$ and P3TEA:PCBM. Since the increasingly negative signal in this region corresponds to growth of EA signal, the kinetics reflect the rate at which free carriers are generated. For the fullerene blend, the signal reaches a plateau within 1ps, while the NFA blend takes ~100 ps to maximize.

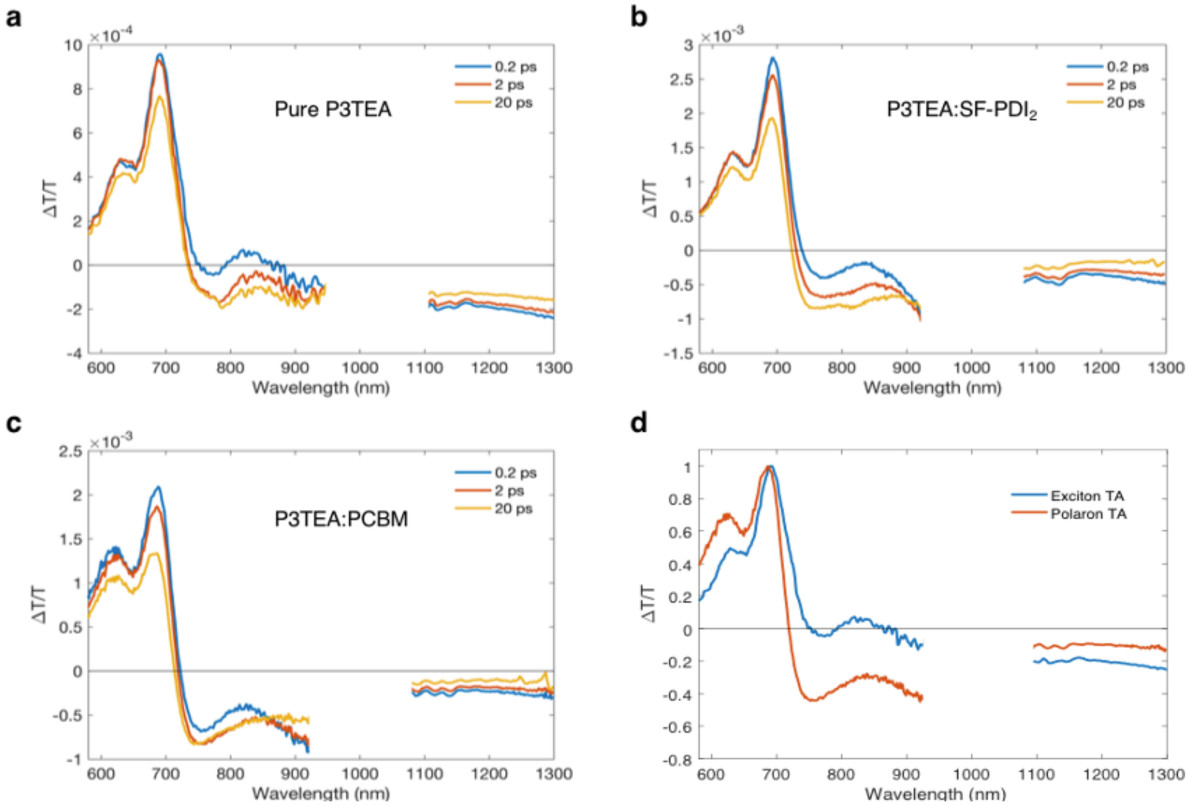

Supplementary Fig. 9. (a,b,c) Un-normalised pump-probe transient absorption (TA) data of pristine P3TEA, P3TEA:SF-PDI$_2$ and P3TEA:PCBM over wide spectral window, excited at 670 nm at ~2 μJ cm^{-2} per pulse. (d) Estimated TA spectrum of P3TEA singlet excitons and polarons (normalised to the peak signal at ~690 nm).

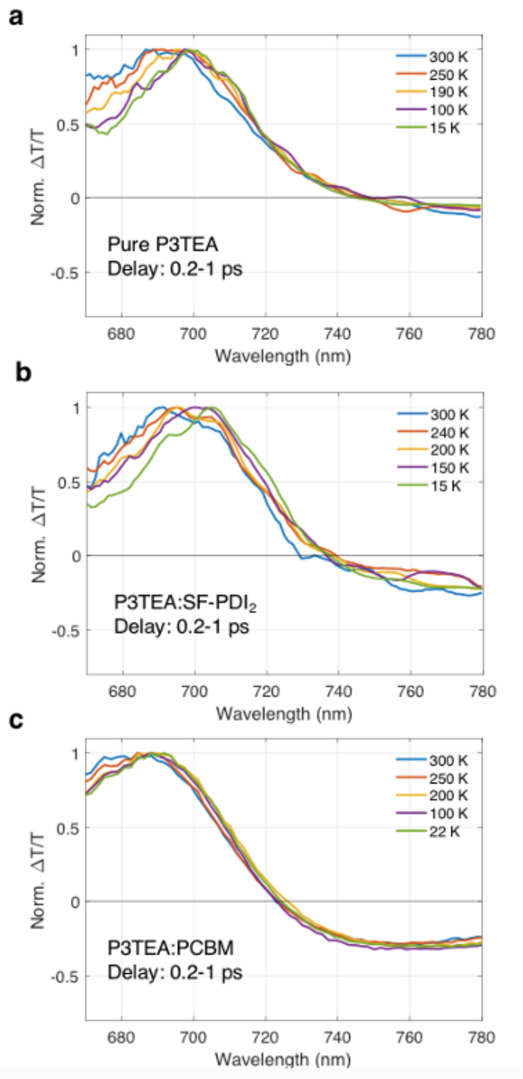

Supplementary Fig. 10. Early-time (0.2-1ps) pump-probe (PP) transient absorption data of (a) P3TEA, (b) P3TEA:SF-PDI_2 and (c) P3TEA:PCBM, all excited at 670nm at a fluence of 1.5 μJ cm^{-2} per pulse, measured at various temperatures. As shown in (a), the pristine P3TEA data show a slight red-shift of the peak ground state bleach (GSB) signal with reducing temperature, which can be explained by lowering of the optical gap. Similar feature is observed in the blend samples upon cooling. (b) TA data for P3TEA:SF-PDI_2 measured at this time range is very similar to the pristine polymer, indicating that excitons make up most of the excited states (charge separation is yet to happen). The small photoinduced absorption (PA) that emerges at ~760nm is likely due to polaron absorption following formation of bound CTEs. (c) For P3TEA:PCBM, the PA signal is even stronger, and the whole spectrum is already blue-shifted with respect to the pure P3TEA film from very early times 0.2-1 ps (crossing zero at ~720nm compared to ~740nm for the other 2 films). As described in main text, this blue-shift is due to emergence of the EA signal (negative from ~710nm onward), thus indicating ultrafast charge separation. Furthermore, this ultrafast separation process is unaffected by temperature (i.e. largely blue-shifted spectrum even at 22K).

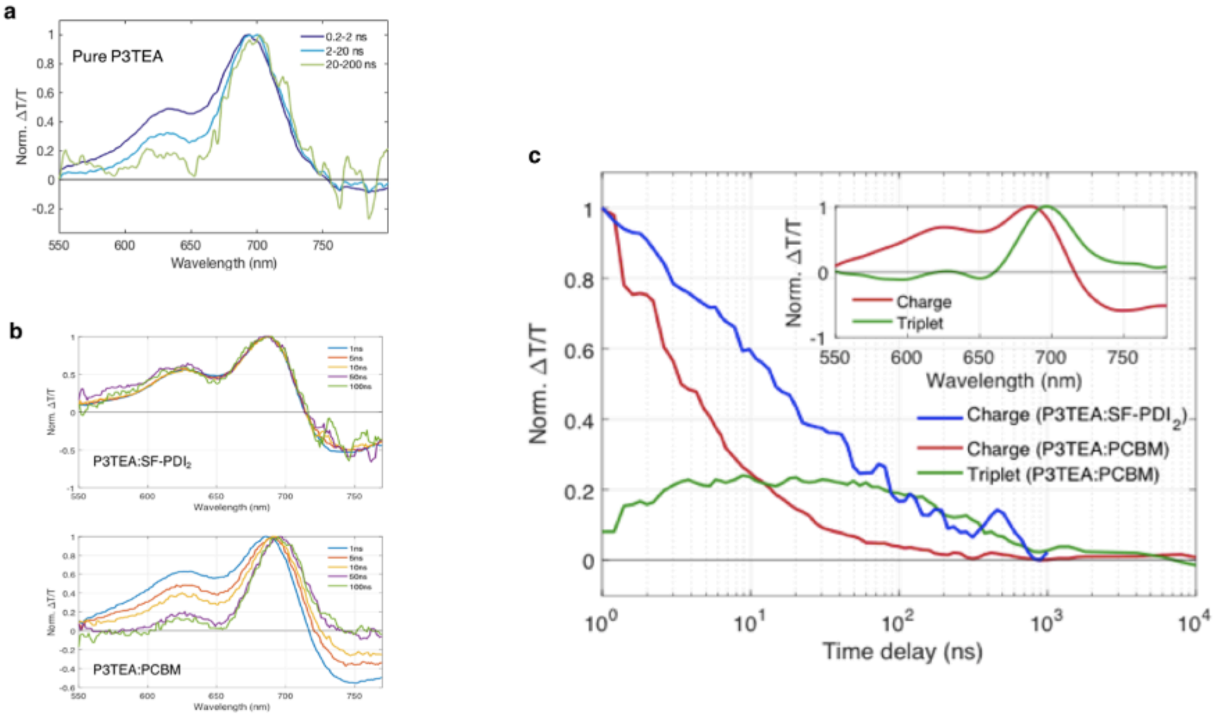

Supplementary Fig. 11. Nanosecond pump-probe transient absorption (TA) spectroscopy. (a) Normalised TA data showing intersystem crossing of P3TEA singlet excitons into triplet excitons at tens of nanoseconds. (b) TA data showing clear spectral shifting on nanosecond timescale due to formation of triplet excitons in P3TEA:PCBM but not in P3TEA:SF-PDI$_2$ (see Supplementary Note 3.3). (c) Charge and triplet exciton signals in P3TEA:PCBM and P3TEA:SF-PDI$_2$ extracted via numerical spectral analysis of TA data. This result suggests that in the P3TEA:PCBM blend charges recombine to form long-lived (~1µs) triplet excitons in the polymer, though additional characterizations are needed to confirm and quantify triplet recombination in this blend. In contrast, we find no spectral shifting in P3TEA:SF-PDI$_2$ over the same timescale, which suggests a suppression of triplet recombination in the non-fullerene blend.

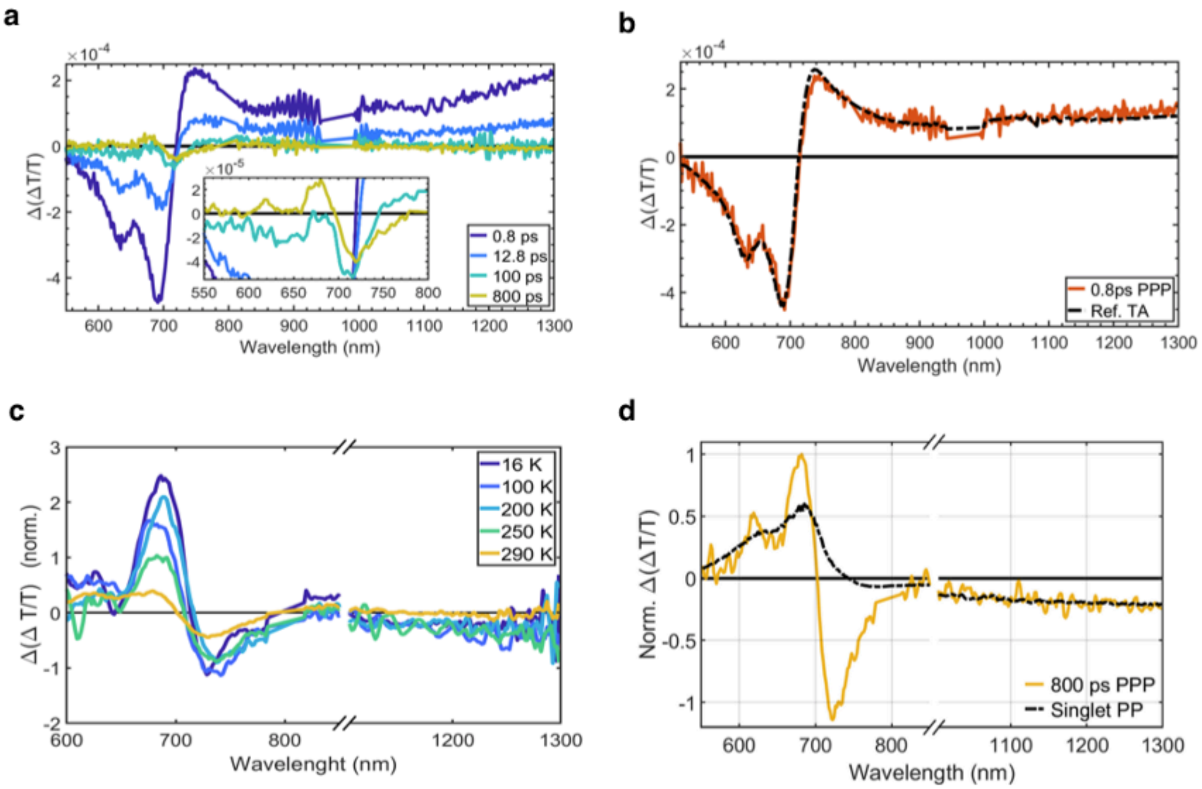

Supplementary Fig. 12. Additional pump-push-probe (PPP) data of P3TEA:SF-PDI_2. (a) PPP signal at pump-push delays from 0.8 to 800 ps, integrated over push-probe delays of 2 to 5ps. The signal is normalised to compensate for changes in the overall excited states population absorbing the push pulse (divided by the pump-probe photoinduced absorption signal near the push wavelength). It therefore shows how the relative contribution of different sub-populations changes. Two domains can be distinguished; At long push delays a derivative-like feature dominates, magnified in the inset. At short push delays the signal corresponds to an inverted pump-probe signal. Subtraction of this 'exciton annihilation' signal reveals the derivative-like electroabsorption signal at long time delays (>50 ns; Figure 3b). (b) Comparison of this PPP signal at short pump-push delays (0.8 ps, with a push-probe delay integrated over 7 to 8 ps) with the inverted and scaled pump-probe data of the same sample at the same pump-probe delay. The Δ(ΔT/T) signal is caused by a reduction in the ΔT/T signal, showing an overall decrease in excited state population. (c) Temperature dependence of pump-push-probe signal of the P3TEA:SF-PDI_2 film (averaged over pump-push delays of 50-100 ps and push-probe delays 0.4-0.5 ps), normalized by the PPP signal in the NIR region at 0.8ps. The signal is decreasing with increasing temperature, particulatly between 250K and 290K, where it is reduced by more than half. (d) Comparison of PPP signal at 800ps with the singlet exciton PP signal taken from a pristine polymer sample. The agreement of the signals in the NIR suggests that at longer pump-push delay times the push can also regenerate singlet excitons.

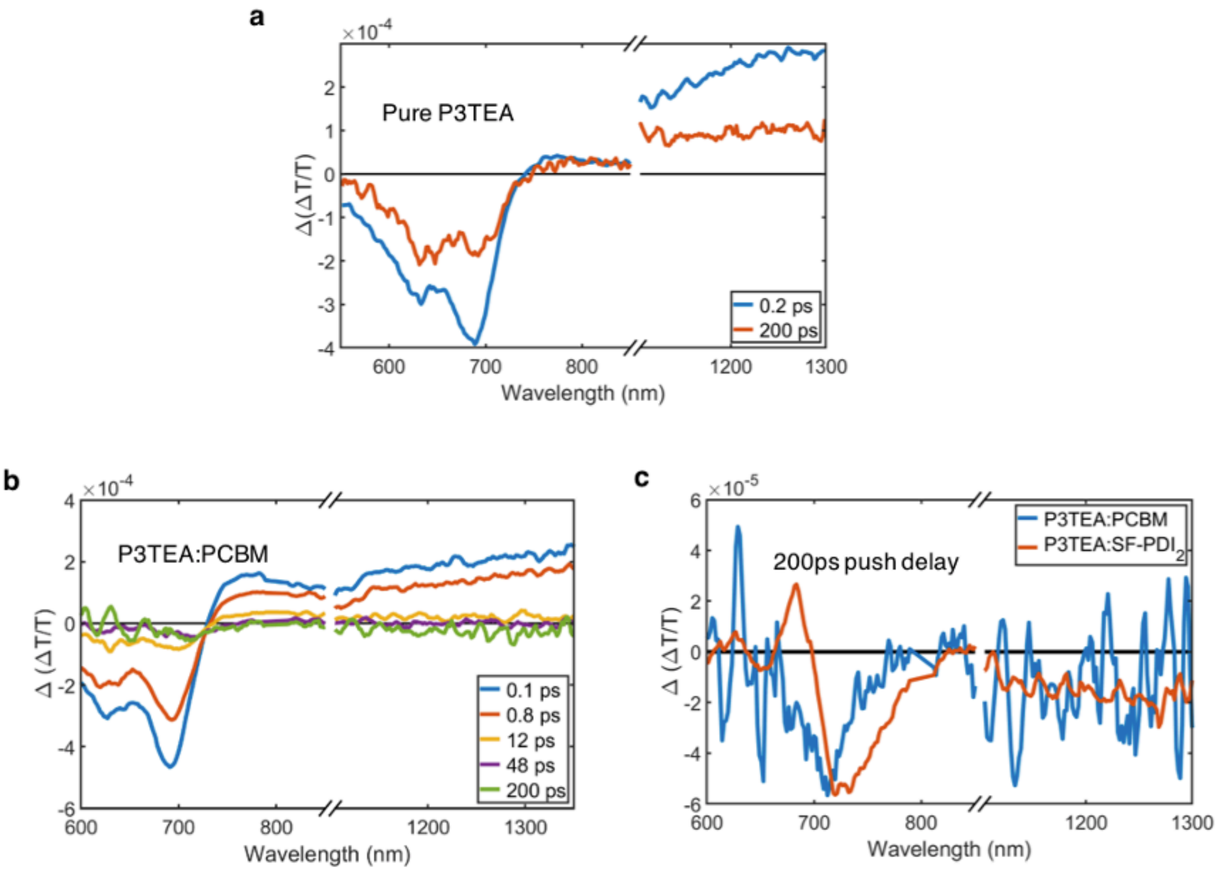

Supplementary Fig. 13. (a) Pump-push-probe data of pristine P3TEA film (normalised and integrated as in Supplementary Fig. 12). At all pump-push delays the signal resembles the inverted pump-probe signal, without a derivative-like signal emerging. (b) Pump-push-probe spectra of the P3TEA:PCBM film at various pump-push delays (normalised and integrated as in Supplementary Fig. 12). At all pump-push delays the signal resembles the inverted pump-probe signal, without a derivative-like signal emerging. (b) Comparison of PPP signal of fullerene-based and non-fullerene films at long pump-push delays (200 ps, with a push-probe delay integrated over 1 to 2 ps). The non-fullerene blend shows the characteristic derivative-like line shape, while the fullerene-based blend does not.

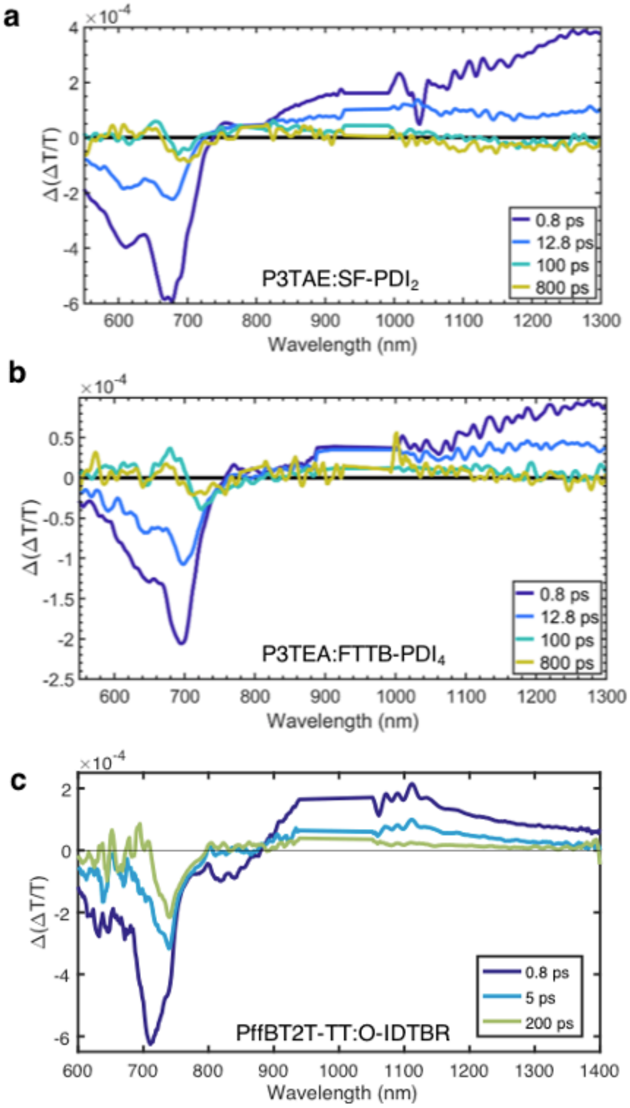

Supplementary Fig. 14. Additional pump-push-probe data of P3TAE:SF-PDI$_2$, P3TEA:FTTB-PDI$_4$ and PffBT2T-TT:O-IDTBR films. (a,b) Data was integrated over push-probe delays of 1 to 2ps and normalised as in Supplementary Fig. 12-13. (c) Data was integrated between 0.5 to 1ps push-probe delays. All blends show similar behaviour: an initial signal that corresponds to an overall reduction of excited states (inverse of ΔT/T signal observed in pristine polymer film), and a longer time signal with a derivative shape near the band edge which corresponds to separation of bound charge-transfer state (CTE) upon push excitation. As in the system discussed in the main text, this derivative-like feature is still present 800ps after the pump, thus indicating a quasi-equilibrium of CTEs and free charges.

Supplementary References

[1] J. Liu, S. Chen, D. Qian, B. Gautam, G. Yang, J. Zhao, J. Bergqvist, F. Zhang, W. Ma, H. Ade, O. Inganäs, K. Gundogdu, F. Gao, H. Yan, *Nat. Energy* **2016**, *1*, 16089.

[2] J. Liu, L.-K. Ma, F. K. Sheong, L. Zhang, H. Hu, J.-X. Zhang, J. Zhang, Z. Li, C. Ma, X. Han, D. Pan, H. Ade, W. Ma, H. Yan, *J. Mater. Chem. A* **2018**, *6*, 16874.

[3] S. Chen, Y. Wang, L. Zhang, J. Zhao, Y. Chen, D. Zhu, H. Yao, G. Zhang, W. Ma, R. H. Friend, P. C. Y. Chow, F. Gao, H. Yan, *Adv. Mater.* **2018**, *30*, 1804215.

[4] J. Zhang, Y. Li, J. Huang, H. Hu, G. Zhang, T. Ma, P. C. Y. Chow, H. Ade, D. Pan, H. Yan, *J. Am. Chem. Soc.* **2017**, *139*, 16092.

[5] S. M. Menke, A. Cheminal, P. Conaghan, N. A. Ran, N. C. Greehnam, G. C. Bazan, T.-Q. Nguyen, A. Rao, R. H. Friend, *Nat. Commun.* **2018**, *9*, 277.

[6] N. A. Ran, J. A. Love, C. J. Takacs, A. Sadhanala, J. K. Beavers, S. D. Collins, Y. Huang, M. Wang, R. H. Friend, G. C. Bazan, T. Q. Nguyen, *Adv. Mater.* **2016**, *28*, 1482.

[7] S. Gelinas, A. Rao, A. Kumar, S. L. Smith, A. W. Chin, J. Clark, T. S. van der Poll, G. C. Bazan, R. H. Friend, *Science.* **2014**, *343*, 512.

[8] F. Gao, W. Tress, J. Wang, O. Inganäs, *Phys. Rev. Lett.* **2015**, *114*, 128701.

[9] A. Rao, P. C. Y. Chow, S. Gélinas, C. W. Schlenker, C.-Z. Li, H.-L. Yip, A. K.-Y. Jen, D. S. Ginger, R. H. Friend, *Nature* **2013**, *500*, 435.

[10] P. C. Y. Chow, S. Gélinas, A. Rao, R. H. Friend, *J. Am. Chem. Soc.* **2014**, *136*, 3424.
